# Supplementary material for: Molecular Abnormalities in BTBR Mice and Their Relevance to Schizophrenia and Autism Spectrum Disorders: An Overview of Transcriptomic and Proteomic Studies
Source: Biomedicines. 2023 Jan 20;11(2):289. doi: 10.3390/biomedicines11020289 (PMC9953015; doi:10.3390/biomedicines11020289)
Supplement: Supplementary file 1 [file biomedicines-11-00289-s001.zip › biomedicines-2140311-supplementary.pdf]

Supplementary Tables

Tab	Title	Caption
Tab 1	Supplementary Table S1a	The list of most reprodusible DE genes in the hippocampus of BTBR mice
Tab 2	Supplementary Table S1b	The list of most reprodusible DE genes in the cortex and striatum of BTBR mice
Tab 3	Supplementary Table S3a	Gene set enrichment analysis of all expressed genes in the hippocampus
Tab 4	Supplementary Table S2b	Gene set enrichment analysis of all expressed genes in the striatum
Tab 5	Supplementary Table S3	The list of "high prediction score" mutations
Tab 6	Supplementary Table S4a	The list of SZH related genes
Tab 7	Supplementary Table S4b	The list of ASD related genes

Supplement Table S1a. The list of most reprodusible DE genes in the hippocampus of BTBR mice

GeneSymbol	Up/down	Number of datasets
Blvrb	up	3
Scg5	up	3
Serpina3n	up	3
Anxa5	up	3
Snapc3	up	2
Clec16a	up	2
Kras	up	2
Nudc	up	2
Cap1	up	2
Uba5	up	2
Adi1	up	2
Mrpl3	up	2
Slc7a14	up	2
Wfs1	up	2
Gins4	up	2
Lpl	up	2
Extl1	up	2
Plcxd2	up	2
Tbc1d9b	up	2
Mamdc2	up	2
Abhd1	up	2
Pla2g4e	up	2
Gbp2b	up	2
Kcnh5	up	2
Mpzl2	up	2
Gon4l	up	2
Wdfy1	up	2
Scly	up	2
Sytl1	up	2
Mroh7	up	2
Exd1	up	2
Cdhr1	up	2
Sfrp1	up	2
Acer2	up	2
Amica1	up	2
Cfap46	up	2
Gabra2	up	2
Folh1	up	2
Hddc3	up	2
Necab3	up	2
C330024D21Rik	up	2
Filip1	up	2
Slc15a2	up	2
2610005L07Rik	up	2
Amy1	up	2
Clcn6	up	2

Me3	up	2
Padi2	up	2
Fv1	up	2
Lrrc27	up	2
Shd	up	2
Ahdc1	up	2
Zfp612	up	2
Ndn	up	2
Faim2	up	2
Wbp11	up	2
C1qb	down	3
Spink8	down	3
Entpd4	down	3
Pop4	down	3
Alg1	down	3
Rpl29	down	3
Ccnd1	down	3
Mt3	down	3
Zfp131	down	3
6330403K07Rik	down	3
Nudt19	down	3
C1ql2	down	3
Evc2	down	3
Cetn4	down	3
1700025G04Rik	down	2
Sv2b	down	2
Ttc17	down	2
Caskin1	down	2
Hsp90b1	down	2
Rgl1	down	2
Armc8	down	2
Clcn7	down	2
Enpp5	down	2
Stk25	down	2
Kcnf1	down	2
Dusp7	down	2
Rapgefl1	down	2
Hsd11b1	down	2
Sema5a	down	2
Crym	down	2
Gnptab	down	2
Hdhd2	down	2
Cort	down	2
Igsf3	down	2
Slc25a3	down	2
Ddhd1	down	2
Zmynd11	down	2
Bdnf	down	2
Anxa3	down	2

Ppm1m	down	2
Lyrm7	down	2
A830018L16Ri k	down	2
Dbndd2	down	2
Bpnt1	down	2
Grhl1	down	2
9330151L19Rik	down	2
Tnni1	down	2
Pgap2	down	2
Ankrd33b	down	2
Trpc6	down	2
Myo7a	down	2
Il1r1	down	2
Myo5b	down	2
Id4	down	2
Zfp846	down	2
Trim12a	down	2
Stpg1	down	2
Thbs4	down	2
Rpl14	down	2
4930503L19Rik	down	2
Tmem260	down	2
Vmn2r29	down	2
C5ar2	down	2
Irgm2	down	2
Tmem254a	down	2
Prr18	down	2
Arhgap15	down	2
Exosc9	down	2
Npy2r	down	2
Lct	down	2
Zfp433	down	2
Trim34a	down	2
Snapc1	down	2
Ptgs2	down	2
Hpgd	down	2
Cml5	down	2
Rec8	down	2
Naaa	down	2
Cisd2	down	2
2900052N01Ri k	down	2
2610044O15Ri k8	down	2
Ctss	down	2
Slc35a5	down	2
Crispld1	down	2
Rps4l	down	2

Krt12	down	2
Gstm6	down	2
Ccbe1	down	2
Eif3j1	down	2
Kctd4	down	2
Frzb	down	2
Thsd4	down	2
Gvin1	down	2
Emp2	down	2
Inmt	down	2
Alox8	down	2
Gm7120	down	2
Gabra5	down	2
Phyh	down	2
Pttg1	down	2
Itpka	down	2
4833420G17Rik	down	2
Ifi203	down	2
Zfp236	down	2
Rint1	down	2
Frrs1l	down	2
Slc37a2	down	2
Mettl10	down	2
Myoc	down	2
Trim30d	down	2
Alox12b	down	2
Gatm	down	2
Eps8l1	down	2
Hjurp	down	2
Acp1	down	2
Tmem159	down	2
Defb11	down	2
Zfp781	down	2
Nxpe4	down	2
Pde6h	down	2
Mobp	down	2
Trf	down	2
Psenen	down	2
Capns1	down	2
Cx3cr1	down	2
Scoc	down	2
Bex4	down	2
Pon2	down	2
Psmb5	down	2
Rnps1	down	2
Pfdn5	down	2
Sepw1	down	2
C1qc	down	2
B2m	down	2
Il33	down	2

Supplement Table S1b. The list of most reproducible DE genes in the cortex and striatum of BTBR mic

GeneSymbol	Up/down	Number of datasets
Scg5	up	4
Serpina3n	up	4
Pla2g4e	up	3
H2-Bl	up	3
Hddc3	up	3
Rwdd3	up	3
Adi1	up	3
Rsph1	up	3
Olfml1	up	3
Ccl17	up	2
5430405H02Rik	up	2
Hemk1	up	2
Anxa4	up	2
Lpl	up	2
Idi2	up	2
Abhd1	up	2
Sytl1	up	2
Masp2	up	2
Cfap54	up	2
Myl1	up	2
AA465934	up	2
A930015D03Rik	up	2
Cap1	up	2
Wdr63	up	2
Gm10704	up	2
Gm5067	up	2
Cox7b2	up	2
Flywch2	up	2
Mns1	up	2
Scn11a	up	2
Lrat	up	2
Gm15459	up	2
Mamdc2	up	2
Gm15487	up	2
Slpi	up	2
Gm15975	up	2
Gm6969	up	2
Gm15631	up	2
Trappc6a	up	2
Gm11942	up	2
Zfp457	up	2
Gm6206	up	2
Capn11	up	2
Gpr15	up	2
Otx2	up	2
Prss41	up	2

Cdhr1	up	2
Sfrp1	up	2
Dydc2	up	2
Ptprv	up	2
2700038G22Rik	up	2
Amd2	up	2
Gm13456	up	2
Myh6	up	2
4930429F24Rik	up	2
Prss56	up	2
Gm10282	up	2
Tmem72	up	2
Plk1	up	2
Gm10060	up	2
Tekt1	up	2
Fam198b	up	2
Rbm46	up	2
4930481B07Rik	up	2
Gm1840	up	2
Scn10a	up	2
Sema3b	up	2
Marveld2	up	2
Folr1	up	2
Meox1	up	2
Sostdc1	up	2
Tnfrsf10b	up	2
Zmynd10	up	2
Fxyd2	up	2
Blvrb	up	2
4930539E08Rik	up	2
B9d1	up	2
Fbxo6	up	2
1190002H23Rik	up	2
C1qb	down	4
Pop4	down	4
Nudt19	down	4
Anxa3	down	4
Slc38a11	down	3
Slc25a34	down	3
Gm14169	down	3
C030034I22Rik	down	3
4930479D17Rik	down	3
Trim12a	down	3
Cela1	down	3
Hist1h2be	down	3

Gm10033	down	3
Gm5148	down	3
Col2a1	down	3
Crispld1	down	3
Hist1h4i	down	3
2610507I01Rik	down	3
Poc1a	down	3
Pttg1	down	3
Tdg	down	3
9030407P20Rik	down	3
Trim30d	down	3
Hjurp	down	3
Krt12	down	3
6330403K07Rik	down	3
Gm12394	down	2
Crocc	down	2
Gpr84	down	2
Ptprc	down	2
Aqr	down	2
Lyrm7	down	2
Mcmdc2	down	2
Thbs4	down	2
Myh7	down	2
Tmem260	down	2
Adgrf4	down	2
Zfp85os	down	2
Zfp984	down	2
Gm10635	down	2
Zfp125	down	2
Gm20208	down	2
2810002D19Rik	down	2
Zfp982	down	2
Adam18	down	2
Frrs1l	down	2
Myoc	down	2
Nanos2	down	2
Gm29514	down	2
Mall	down	2
Tmem159	down	2
Stac2	down	2
Scoc	down	2
Rbbp9	down	2
Alg1	down	2
Rpp25	down	2
Fgfr1op2	down	2
Mt3	down	2
Slc25a3	down	2
Zmynd11	down	2

Mmp28	down	2
9330104G04Rik	down	2
Fcrls	down	2
1700010I14Rik	down	2
9330151L19Rik	down	2
Tnni1	down	2
Sfn	down	2
Tubb6	down	2
Myo7a	down	2
4930503L19Rik	down	2
Pglyrp1	down	2
Sema3g	down	2
Gm9866	down	2
Pdlim2	down	2
Impg1	down	2
Ncf2	down	2
Hpgd	down	2
B230217O12Rik	down	2
A830036E02Rik	down	2
Rec8	down	2
Col6a4	down	2
Cmya5	down	2
Gm5083	down	2
Arhgef19	down	2
Lmod1	down	2
Slfn8	down	2
Gstm6	down	2
Nkx2-9	down	2
Gpr160	down	2
Sp110	down	2
Alox8	down	2
Myl6b	down	2
4833420G17Rik	down	2
Ifi203	down	2
Oscar	down	2
Slc37a2	down	2
Eps8l1	down	2
Gm6970	down	2
Hfm1	down	2
Darc	down	2
C1qc	down	2
B2m	down	2
Ddit4l	down	2
Crym	down	2
Il33	down	2

Mobp	down	2
Myl4	down	2
Rnps1	down	2
Rpl29	down	2
Hist1h2ao	down	2
Tnnc1	down	2
Cort	down	2
Cox6a2	down	2

Supplement Table S2a. Gene set enrichment analysis of all expressed genes in the hippocampus

NTOLOG	ID	Description	setSize	richmentSc	NES	pvalue	p.adjust	qvalues	rank	ading_edge_enrichment
BP	GO:00076	sensory perception of chemical s	343	0.604539	1.88241	1E-10	1E-10	6.3E-07	2431	tags=50%, Olfr788/Olfr691/Olfr720/Olfr1039/Olfr575/Olfr25/Olfr30/Olfr482/Olfr14 [illegible]
MF	GO:00049	olfactory receptor activity	293	0.619931	1.91127	1.2943E-10	1.2943E-10	6.3E-07	2431	tags=54%, Olfr788/Olfr691/Olfr720/Olfr1039/Olfr575/Olfr25/Olfr30/Olfr482/Olfr14 [illegible]
BP	GO:00076	sensory perception of smell	305	0.608177	1.88298	1.3007E-10	1.3007E-10	6.3E-07	2920	tags=56%, Olfr788/Olfr691/Olfr720/Olfr1039/Olfr575/Olfr25/Olfr30/Olfr482/Olfr14 [illegible]
BP	GO:00076	sensory perception	757	0.483407	1.56594	5.3071E-08	5.3071E-08	0.00019	2920	tags=35%, Olfr788/Lrat/Pde6g/Scn11a/Tulp1/Olfr691/Slc45a2/Olfr720/Olfr1039/Olf [illegible]
MF	GO:00055	odorant binding	126	0.64458	1.83061	2.361E-06	2.361E-06	0.00683	3052	tags=62%, Olfr1039/Olfr25/Vmn1r38/Olfr482/Olfr1432/Olfr922/Olfr1090/Vmn1r78 [illegible]
BP	GO:00514	corticotropin secretion	10	-0.88566	-1.77658	0.00100461	0.00100461	1	261	tags=40%, Apln/Ecrg4/Aqp1/Lif
BP	GO:00514	regulation of corticotropin secre	8	-0.91729	-1.75348	0.00101305	0.00101305	1	261	tags=38%, Apln/Ecrg4/Lif
BP	GO:00514	positive regulation of corticotrop	7	-0.92227	-1.714	0.00116544	0.00116544	1	261	tags=43%, Apln/Ecrg4/Lif
BP	GO:00070	mitotic sister chromatid cohesion	27	-0.75763	-1.87585	0.00124795	0.00124795	1	1093	tags=22%, Cdca5/Cdc20/Smc1b/Dscc1/Rec8/Pttg1
BP	GO:00974	protein maturation by iron-sulfu	14	0.834308	1.66726	0.00273043	0.00273043	1	8	tags=7%, l AK157302
BP	GO:00076	phototransduction, visible light	9	0.873287	1.59802	0.00330674	0.00330674	1	697	tags=44%, Aipl1/Pde6c/Grk1/Pcp2
BP	GO:00192	response to pheromone	49	0.65095	1.64325	0.00396806	0.00396806	1	3060	tags=65%, Vmn1r38/Vmn1r50/Vmn1r78/Vmn1r226/Vmn1r193/Vmn1r9/Vmn1r229 [illegible]
MF	GO:00706	activin receptor binding	9	-0.86449	-1.68603	0.00429508	0.00429508	1	1771	tags=44%, Smad6/Nodal/Gdf9/Tdgf1
MF	GO:00021	aminoacyl-tRNA editing activity	13	-0.82448	-1.73522	0.00438164	0.00438164	1	17	tags=15%, Iars2/Lars2
MF	GO:00055	mannose binding	19	0.79816	1.70623	0.00451575	0.00451575	1	2050	tags=37%, Colec11/Mbl2/Cd209a/Clec4g/Clec4n/Cd209b/Cd209c
BP	GO:19015	regulation of endothelial tube m	5	-0.93073	-1.62131	0.00504629	0.00504629	1	1050	tags=80%, Adamts12/Itgax/Alox12/Cxcl10
MF	GO:00317	CCR6 chemokine receptor bindi	4	-0.95396	-1.57045	0.00566779	0.00566779	1	18	tags=50%, Defb10/Defb1
BP	GO:00450	positive regulation of innate imm	102	-0.50094	-1.55773	0.00596339	0.00596339	1	2797	tags=36%, Mavs/Srebf1/Lag3/Nod2/Lgals9/Polr3f/Irgm1/Aim2/Txk/Tlr2/Card9/Tyro [illegible]
BP	GO:00108	regulation of T cell chemotaxis	12	-0.814	-1.67331	0.00607686	0.00607686	1	1359	tags=33%, Ccr2/Ccr7/Cxcl10/Tmem102
MF	GO:00526	carboxylic ester hydrolase activi	120	-0.46179	-1.43841	0.00627212	0.00627212	1	1492	tags=20%, Abhd12b/Acot2/Abhd4/Pon2/Rpe65/Acot12/Lipi/Aspg/Pla2g2f/Oc90/Ba [illegible]
BP	GO:01501	regulation of transepithelial tran	4	-0.9494	-1.56296	0.00705226	0.00705226	1	332	tags=75%, Actb/Cldn19/Cldn3
MF	GO:00170	U6 snRNA binding	12	-0.80975	-1.66458	0.00722522	0.00722522	1	112	tags=33%, Prpf4/Rbm22/Larp7/Tex16
BP	GO:00724	ammonium transmembrane trans	5	-0.92037	-1.60325	0.00794237	0.00794237	1	220	tags=50%, Rhcg/Rhbg/Rhd
BP	GO:00311	animal organ regeneration	26	-0.69879	-1.7242	0.00823558	0.00823558	1	2313	tags=35%, Apoa1/Egfr/Cebpb/Cdk1/Ccnd1/Ace/Cxcl5/Apoa2/Lif
BP	GO:00108	T cell chemotaxis	17	-0.76228	-1.70089	0.00875637	0.00875637	1	1359	tags=53%, Cxcl12/Adam17/Slc12a2/Ccr2/Cxcr3/Cxcl16/Ccr7/Cxcl10/Tmem102
MF	GO:00165	pheromone receptor activity	44	0.650292	1.61578	0.00910655	0.00910655	1	3060	tags=68%, Vmn1r38/Vmn1r50/Vmn1r78/Vmn1r226/Vmn1r193/Vmn1r9/Vmn1r229 [illegible]
BP	GO:00313	positive regulation of defense re	228	-0.38468	-1.31518	0.00954232	0.00954232	1	2627	tags=29%, Ctsc/Lag3/Nod2/Lgals9/Tac1/Polr3f/Mdk/Irgm1/Cyba/Tlr3/Npy5r/Aim2/ [illegible]
BP	GO:00714	cellular response to salt stress	13	-0.79564	-1.67453	0.00956819	0.00956819	1	472	tags=36%, Capn3/Efhd1/Trpv4/Aqp1/Slc12a1
MF	GO:00706	transmembrane receptor protein	22	-0.70565	-1.67656	0.00994384	0.00994384	1	1771	tags=36%, Smad6/Pycard/Bmp8b/Acvr1c/Nodal/Gdf9/Gdf15/Tdgf
BP	GO:20004	positive regulation of T cell mig	28	-0.67793	-1.68777	0.01017954	0.01017954	1	3415	tags=39%, Selenok/Cxcl12/Itga4/Adam17/Lgals9/Aif1/Pycard/Ccr2/Ccr7/Cxcl10/Tm [illegible]
BP	GO:00162	iron-sulfur cluster assembly	23	0.73489	1.62831	0.01067681	0.01067681	1	8	tags=4%, l AK157302
BP	GO:00311	metallo-sulfur cluster assembly	23	0.73489	1.62831	0.01067681	0.01067681	1	8	tags=4%, l AK157302
MF	GO:00190	GMP binding	4	0.944257	1.50719	0.01069857	0.01069857	1	33	tags=25%, Gbp2b
BP	GO:00070	sister chromatid cohesion	54	-0.58415	-1.64726	0.0108248	0.0108248	1	1093	tags=19%, Cdca5/Kif22/Cdc20/Esco2/Smc1b/Ddx11/Dscc1/Bub1b/Rec8/Pttg1
BP	GO:00026	regulation of leukocyte migratio	193	-0.40779	-1.37156	0.01137513	0.01137513	1	2743	tags=25%, Pecam1/Pdgfd/Thbs1/Il1a/Nod2/Lgals9/Csf1/Mdk/Pla2g7/Cd9/Tlr2/Cd30 [illegible]
MF	GO:00701	small ribosomal subunit rRNA b	10	-0.82127	-1.64741	0.01160853	0.01160853	1	66	tags=40%, Utp23/Mrps18c/Rps3/Rps13
MF	GO:00080	high-density lipoprotein particle	11	-0.82346	-1.68213	0.01167457	0.01167457	1	2355	tags=55%, Cd36/Apoa1/Trem2/Gpihbp1/Apoa2/Pon1
MF	GO:00336	receptor serine/threonine kinase	29	-0.64184	-1.61248	0.01182349	0.01182349	1	1771	tags=28%, Smad6/Pycard/Bmp8b/Acvr1c/Nodal/Gdf9/Gdf15/Tdgf
BP	GO:00096	response to salt stress	25	-0.67357	-1.66165	0.01199041	0.01199041	1	472	tags=27%, Capn3/Nedd4l/Efhd1/Trpv4/Kmo/Aqp1/Slc12a1
MF	GO:00085	ammonium transmembrane trans	8	-0.85795	-1.64004	0.01237594	0.01237594	1	220	tags=44%, Slc12a2/Rhbg/Rhd/Aqp1
BP	GO:00156	ammonium transport	8	-0.85795	-1.64004	0.01237594	0.01237594	1	220	tags=44%, Slc12a2/Rhbg/Rhd/Aqp1
MF	GO:01501	RNA binding involved in posttra	3	0.961908	1.45829	0.01258547	0.01258547	1	127	tags=33%, Zar1l
MF	GO:19032	mRNA binding involved in post	3	0.961908	1.45829	0.01258547	0.01258547	1	127	tags=33%, Zar1l
BP	GO:00327	positive regulation of chemokine	58	-0.56555	-1.61289	0.01275072	0.01275072	1	3445	tags=50%, Cd84/Selenok/Postn/Adam17/Il16/Hc/Mavs/Il18/Il1a/Mbp/Nod2/Lgals9/T [illegible]
MF	GO:00081	S-methyltransferase activity	6	-0.89057	-1.62661	0.013079	0.013079	1	450	tags=50%, Mgmt/Inmt/Bhmt
BP	GO:20004	regulation of T cell migration	39	-0.60127	-1.60505	0.01367408	0.01367408	1	1716	tags=23%, Aif1/Pycard/Ccr2/Il27ra/Lrch1/Ccr7/Aire/Cxcl10/Tmem102
BP	GO:00148	response to stimulus involved in	12	0.795275	1.5475	0.01423103	0.01423103	1	1542	tags=33%, Myog/Prkag3/Trim63/Actn3
BP	GO:00726	T cell migration	52	-0.54792	-1.53689	0.01512667	0.01512667	1	1735	tags=29%, Icam1/Aif1/Pycard/Ccr2/Cxcr3/F11r/Il27ra/Cxcl16/Myo1g/Lrch1/Ccr7/A [illegible]
BP	GO:00420	T-helper 1 type immune respons	43	-0.5612	-1.5079	0.01521089	0.01521089	1	2137	tags=33%, Il12rb1/Slamf1/Slc11a1/Tmem98/Ccl19/Ccr2/Il33/Il18rap/Nlrp10/Il27ra/ [illegible]
CC	GO:00442	C-fiber	4	0.935081	1.49254	0.01681135	0.01681135	1	1210	tags=100% Scn11a/Scn10a/Ccl2/Prph
BP	GO:00096	response to virus	259	-0.37623	-1.30812	0.01755978	0.01755978	1	2576	tags=24%, Lgals9/Polr3f/Tspan32/Ticam2 [illegible]
BP	GO:00308	prostate gland development	48	-0.57404	-1.58155	0.01769735	0.01769735	1	1182	tags=21%, Stat5a/Esr2/Rln1/Cyp19a1/N [illegible]
BP	GO:00075	body fluid secretion	79	-0.48836	-1.45977	0.01785971	0.01785971	1	2566	tags=30%, Ptger4/Tifab/Nme1/Socs2/F [illegible]
BP	GO:00380	nodal signaling pathway	15	-0.72953	-1.58453	0.01799029	0.01799029	1	2650	tags=47%, Tgif2/Tgif1/Lefty1/Acvr1c/N [illegible]
MF	GO:00040	aryl sulfotransferase activity	3	-0.95938	-1.49645	0.01849281	0.01849281	1	77	tags=67%, Sult1c1/Sult1c2
BP	GO:00321	positive regulation of response t	359	-0.35468	-1.26366	0.01851761	0.01851761	1	2711	tags=26%, Napepld/Pdgfd/Thbs1/Ctsc/ [illegible]
CC	GO:00451	apical part of cell	412	-0.33999	-1.21942	0.01851765	0.01851765	1	2450	tags=22%, Atp8b1/Erbb3/Fxyd1/Cyba/ [illegible]
CC	GO:00349	nuclear mitotic cohesin complex	3	-0.95874	-1.49546	0.0191592	0.0191592	1	40	tags=67%, Rad21l/Rec8
MF	GO:00905	phosphatidylethanolamine flippa	7	-0.8542	-1.58749	0.01938029	0.01938029	1	1797	tags=43%, Atp11c/Atp8b5/Abca4
BP	GO:00022	activation of innate immune resp	36	-0.60184	-1.57708	0.01948532	0.01948532	1	2234	tags=36%, Aim2/Tlr2/Tyrobp/Ifi208/Py [illegible]
BP	GO:00160	rhodopsin mediated signaling pa	7	0.866931	1.51885	0.0199392	0.0199392	1	697	tags=43%, Aipl1/Grk1/Pcp2
BP	GO:19049	negative regulation of leukocyte	4	-0.9288	-1.52904	0.02009925	0.02009925	1	12	tags=50%, Cxcl12/Ccl28
BP	GO:00072	osmosensory signaling pathway	3	-0.9574	-1.49335	0.02015878	0.02015878	1	472	tags=67%, Oxsr1/Trpv4
MF	GO:00316	G-protein beta/gamma-subunit c	22	-0.66799	-1.58709	0.02074724	0.02074724	1	1905	tags=27%, Gna15/Gna14/Plcb2/Cetn4/ [illegible]
BP	GO:19032	negative regulation of leukocyte	3	-0.95634	-1.4917	0.02082517	0.02082517	1	12	tags=67%, Cxcl12/Ccl28
BP	GO:00757	intracellular transport of virus	4	-0.92698	-1.52605	0.02114788	0.02114788	1	68	tags=50%, Fmr1/Kpna2
BP	GO:00026	negative regulation of leukocyte	39	-0.58821	-1.57019	0.02128413	0.02128413	1	1192	tags=21%, Il33/Il27ra/Cyp19a1/Lrch1/ [illegible]
BP	GO:00722	metanephric nephron epithelium	16	-0.73344	-1.6171	0.02134517	0.02134517	1	2335	tags=50%, Hes1/Lgr4/Calb1/Hes5/Wwt [illegible]
CC	GO:00331	proton-transporting two-sector A	15	0.753566	1.54045	0.02166979	0.02166979	1	858	tags=13%, Atp6v1e2/Atp6v1c2
BP	GO:00069	response to osmotic stress	71	-0.49271	-1.44777	0.0219551	0.0219551	1	2143	tags=17%, Efhd1/Slc12a2/Serpinb6a/M [illegible]
BP	GO:00722	metanephric nephron tubule dev	14	-0.75035	-1.62375	0.02199016	0.02199016	1	2335	tags=50%, Hes1/Lgr4/Calb1/Hes5/Wwt [illegible]
BP	GO:00706	transepithelial transport	24	-0.66111	-1.59757	0.02207017	0.02207017	1	970	tags=29%, Slc12a2/Abcg2/Slc5a1/Cldn [illegible]
BP	GO:00427	very long-chain fatty acid biosyn	12	-0.76902	-1.58085	0.02222863	0.02222863	1	1079	tags=17%, Elovl7/Tecrl
BP	GO:00065	cellular amino acid metabolic pr	227	-0.38112	-1.30035	0.02285994	0.02285994	1	2560	tags=16%, Sds/Cth/Ivd/Ido2/Cars/Srr/P [illegible]
BP	GO:00025	positive regulation of antigen pr	10	-0.79558	-1.59588	0.02297551	0.02297551	1	2587	tags=60%, Nod2/Slc11a1/Ccl19/Trem2/ [illegible]
MF	GO:00706	type I activin receptor binding	5	-0.88971	-1.54984	0.02322113	0.02322113	1	1771	tags=80%, Smad6/Nodal/Gdf9/Tdgf1
CC	GO:00312	intrinsic component of the cytop	4	0.924411	1.47551	0.02332726	0.02332726	1	134	tags=25%, Nphs2
BP	GO:00714	cellular response to osmotic stre	36	-0.58937	-1.5444	0.02341517	0.02341517	1	2143	tags=24%, Efhd1/Serpinb6a/Mylk/Slc2 [illegible]
MF	GO:00198	growth factor binding	139	-0.41571	-1.32779	0.02346682	0.02346682	1	1986	tags=20%, Egfr/Igfbp7/Itgb4/Ghr/Hyal2 [illegible]
CC	GO:00430	costamere	19	-0.70157	-1.59975	0.02353171	0.02353171	1	843	tags=21%, Cmya5/Smpx/Krt8/Krt19
MF	GO:00718	lipoprotein particle binding	27	-0.65758	-1.62813	0.02359052	0.02359052	1	2681	tags=37%, Thbs1/Cd36/Apoa1/Scarf1/M [illegible]
MF	GO:00718	protein-lipid complex binding	27	-0.65758	-1.62813	0.02359052	0.02359052	1	2681	tags=37%, Thbs1/Cd36/Apoa1/Scarf1/M [illegible]
BP	GO:00607	epithelial cell differentiation inv	12	-0.76668	-1.57603	0.02377493	0.02377493	1	1182	tags=42%, Ctnnb1/Fgfr2/Stat5a/Esr2/Pl [illegible]
CC	GO:00315	brush border membrane	68	-0.504	-1.48133	0.02415315	0.02415315	1	2450	tags=29%, Atp8b1/Cd36/Atp7a/Abcg2/ [illegible]
MF	GO:00317	CCR2 chemokine receptor bindi	4	0.921984	1.47163	0.02436981	0.02436981	1	1159	tags=50%, Ccl2/Ccl7
BP	GO:00148	response to muscle activity invo	5	0.898228	1.48357	0.02437565	0.02437565	1	376	tags=40%, Myog/Prkag3
BP	GO:20004	regulation of lymphocyte migrat	51	-0.53074	-1.49017	0.02471977	0.02471977	1	1716	tags=20%, Aif1/Pycard/Ccr2/Il27ra/Lrc [illegible]
CC	GO:00988	cluster of actin-based cell projec	180	-0.39478	-1.3192	0.02486925	0.02486925	1	1931	tags=22%, Rhoc/Atp8b1/Cd36/Myo6/C [illegible]
BP	GO:00183	protein farnesylation	4	0.920423	1.46914	0.02515172	0.02515172	1	168	tags=25%, Aipl1
BP	GO:00607	positive regulation of response t	52	-0.52568	-1.47451	0.02515345	0.02515345	1	4286	tags=47%, Ikbke/Dhx9/Ifih1/Ube2k/Rip [illegible]
BP	GO:00716	positive regulation of mononucle	50	-0.52243	-1.45114	0.02515345	0.02515345	1	3202	tags=36%, Selenok/Cxcl12/Itga4/Adam [illegible]
BP	GO:00600	synaptic transmission, glycinerg	7	0.856715	1.50095	0.02527318	0.02527318	1	1660	tags=57%, Glra4/Slc6a5/Glra1/Glra3
BP	GO:00070	cell-cell junction assembly	120	-0.42719	-1.33064	0.02533689	0.02533689	1	2803	tags=25%, Fzd5/Tbcd/Pecam1/Cldn34c [illegible]
BP	GO:00433	negative T cell selection	14	-0.74483	-1.61182	0.02537319	0.02537319	1	2190	tags=43%, Zap70/Ptprc/Cd28/Themis/C [illegible]
MF	GO:00705	metallodipeptidase activity	3	-0.95193	-1.48483	0.02548992	0.02548992	1	897	tags=100% Ace/Dpep1
BP	GO:00327	positive regulation of interleukin	53	-0.52086	-1.47119	0.025802	0.025802	1	2888	tags=38%, Il16/Fzd5/Nod2/Lgals9/Aim [illegible]
MF	GO:00080	ATP-dependent activity, acting	51	-0.52412	-1.47159	0.02600946	0.02600946	1	2690	tags=22%, Chtf8/Rfc5/Btaf1/Blm/Rad5 [illegible]
BP	GO:00708	bicellular tight junction assembl	56	-0.50547	-1.43171	0.02652147	0.02652147	1	2937	tags=36%, Nedd4l/Fzd5/Tbcd/Pecam1/ [illegible]
BP	GO:00523	modulation by symbiont of entry	49	-0.53045	-1.46844	0.0267468	0.0267468	1	937	tags=18%, Ifitm6/Triml2/Ciita/Ifitm7/T [illegible]
BP	GO:00001	sulfur amino acid transport	4	-0.92016	-1.51482	0.02709014	0.02709014	1	149	tags=50%, Slc1a1/Slc3a1
BP	GO:00422	cellular modified amino acid cat	20	-0.67299	-1.55989	0.0272187	0.0272187	1	2084	tags=35%, Abhd12b/Dio3/Chac1/Ggt1/ [illegible]
BP	GO:00026	negative regulation of cellular ex	8	-0.8218	-1.57094	0.02741595	0.02741595	1	996	tags=50%, Ptger4/Cxcl12/Il27ra/Ccl28
BP	GO:00029	negative regulation of humoral i	10	-0.78715	-1.57898	0.02760614	0.02760614	1	3613	tags=80%, Serping1/Cr1l/Cd59b/Foxj1/ [illegible]
BP	GO:00516	defense response to virus	211	-0.38235	-1.29929	0.02791357	0.02791357	1	2531	tags=24%, Polr3f/Tspan32/Ticam2/Il10 [illegible]
BP	GO:01405	defense response to symbiont	211	-0.38235	-1.29929	0.02791357	0.02791357	1	2531	tags=24%, Polr3f/Tspan32/Ticam2/Il10 [illegible]
MF	GO:00162	CoA hydrolase activity	21	-0.65348	-1.53826	0.02845181	0.02845181	1	2899	tags=48%, Acaa2/Acot6/Acot2/Acot12/ [illegible]
MF	GO:00161	amylase activity	4	0.917425	1.46436	0.02855028	0.02855028	1	1538	tags=100% Mgam/Amy2a5/Amy2a4/Am [illegible]
BP	GO:00026	positive regulation of leukocyte	131	-0.42012	-1.31915	0.02866386	0.02866386	1	4066	tags=39%, Akirin1/Ptafr/Jam3/Pawr/Itg [illegible]
BP	GO:00148	response to inactivity	6	0.856618	1.46267	0.02887292	0.02887292	1	1542	tags=50%, Myog/Trim63/Actn3
BP	GO:00148	response to muscle inactivity	6	0.856618	1.46267	0.02887292	0.02887292	1	1542	tags=50%, Myog/Trim63/Actn3
BP	GO:00148	response to muscle inactivity inv	6	0.856618	1.46267	0.02887292	0.02887292	1	1542	tags=50%, Myog/Trim63/Actn3
BP	GO:00148	response to denervation involve	6	0.856618	1.46267	0.02887292	0.02887292	1	1542	tags=50%, Myog/Trim63/Actn3
CC	GO:00308	meiotic cohesin complex	7	-0.83938	-1.55996	0.02916077	0.02916077	1	2095	tags=43%, Stag3/Smc1b/Rec8
MF	GO:00084	CoA-transferase activity	6	0.856332	1.46218	0.02939314	0.02939314	1	1629	tags=50%, Oxct2a/Acsm3/Oxct2b
BP	GO:00390	pronephric nephron developmen	3	-0.94648	-1.47633	0.02948829	0.02948829	1	215	tags=67%, Ahi1/Hnf1b
BP	GO:00022	microglial cell activation involv	5	-0.88002	-1.53296	0.03031143	0.03031143	1	1951	tags=80%, Cx3cr1/Tyrobp/Trem2/Il33
MF	GO:00043	fatty-acyl-CoA synthase activity	4	0.914523	1.45973	0.03064566	0.03064566	1	1391	tags=75%, Acsm5/Acsm4/Acsm3
BP	GO:00518	cytolysis in other organism invo	3	0.944402	1.43175	0.03072194	0.03072194	1	778	tags=67%, Camp/F2
BP	GO:00518	cytolysis by host of symbiont ce	3	0.944402	1.43175	0.03072194	0.03072194	1	778	tags=67%, Camp/F2
BP	GO:00336	positive regulation of cell-cell ad	5	0.884884	1.46153	0.03084706	0.03084706	1	504	tags=20%, Skap1
MF	GO:00427	PH domain binding	5	0.885115	1.46191	0.03084706	0.03084706	1	96	tags=20%, Samd11
BP	GO:00068	phosphate ion transport	23	-0.62651	-1.49886	0.03089296	0.03089296	1	1457	tags=30%, Atf4/Slc25a30/Cebpb/Stc2/S [illegible]
CC	GO:00308	mitotic cohesin complex	4	-0.9144	-1.50533	0.03093514	0.03093514	1	40	tags=50%, Sgo2a/Rec8
BP	GO:00028	regulation of T-helper 1 type im	25	-0.62991	-1.55394	0.03103968	0.03103968	1	2947	tags=48%, Pla2g4a/Il18/Il12rb1/Slc11a [illegible]
CC	GO:00226	cytosolic small ribosomal subun	44	-0.54359	-1.45651	0.03153068	0.03153068	1	3695	tags=18%, Rps26/Eif2a/Ddx3x/Mcts1/ [illegible]
BP	GO:00716	regulation of mononuclear cell m	94	-0.45767	-1.41185	0.03177294	0.03177294	1	3481	tags=33%, C3ar1/Selenok/Msn/Cxcl12/ [illegible]
BP	GO:00000	mitotic sister chromatid segregat	151	-0.39442	-1.29167	0.03240511	0.03240511	1	2671	tags=15%, Pcid2/Nusap1/Incenp/Ndc80 [illegible]
MF	GO:00515	2 iron, 2 sulfur cluster binding	26	0.676413	1.52912	0.0326426	0.0326426	1	600	tags=8%, l AK157302/Aox4
BP	GO:00059	disaccharide metabolic process	4	0.910776	1.45374	0.03274104	0.03274104	1	1276	tags=50%, Fbp2/Fbp1
BP	GO:00148	response to electrical stimulus in	3	0.942243	1.42848	0.03289689	0.03289689	1	1018	tags=67%, Myog/Trim63
BP	GO:00313	regulation of defense response	497	-0.32045	-1.16035	0.03326603	0.03326603	1	2656	tags=23%, Fanca/Ctsc/Lag3/Nod2/Lgal [illegible]
BP	GO:19024	regulation of histone H3-K27 tri	3	0.940593	1.42598	0.03398437	0.03398437	1	144	tags=33%, Ezhip
BP	GO:00432	apical junction assembly	65	-0.49102	-1.44356	0.03404384	0.03404384	1	2937	tags=32%, Nedd4l/Fzd5/Tbcd/Pecam1/ [illegible]
BP	GO:00065	S-adenosylmethionine biosynthe	4	-0.91083	-1.49945	0.03408106	0.03408106	1	317	tags=50%, Mettl16/Mat1a
BP	GO:00465	regulation of viral entry into hos	44	-0.53967	-1.446	0.03414008	0.03414008	1	937	tags=34%, Trim26/Lgals1/Triml1/Trim [illegible]
MF	GO:00055	pheromone binding	38	0.619655	1.50798	0.03453269	0.03453269	1	3052	tags=66%, Vmn1r38/Vmn1r78/Vmn1r2 [illegible]
CC	GO:00059	brush border	125	-0.42333	-1.32663	0.03454811	0.03454811	1	2450	tags=22%, Atp8b1/Cd36/Myo6/Atp7a/S [illegible]
BP	GO:00717	response to nitric oxide	16	-0.70755	-1.56002	0.03557497	0.03557497	1	3001	tags=31%, Cflar/Thbs1/Ccl19/Ccr7/Aq [illegible]
BP	GO:00714	cellular hyperosmotic response	11	-0.78137	-1.59615	0.03581395	0.03581395	1	176	tags=27%, Efhd1/Aqp1/Slc12a1
BP	GO:00108	positive regulation of cell-substr	129	-0.40519	-1.26487	0.03601681	0.03601681	1	2896	tags=22%, Sdc4/Rras/Disc1/Has2/Thbs1 [illegible]
BP	GO:00517	biological process involved in in	133	-0.3948	-1.24619	0.03606093	0.03606093	1	2647	tags=20%, Thoc6/Trim26/Lgals1/Irgm1 [illegible]
BP	GO:00381	ERBB2 signaling pathway	3	-0.93978	-1.46588	0.03649857	0.03649857	1	827	tags=67%, Cpne3/Myoc
BP	GO:00019	positive regulation of cell-matrix	57	-0.49688	-1.40549	0.03693724	0.03693724	1	2848	tags=21%, Rras/Disc1/Plekha2/Csf1/Cd [illegible]
BP	GO:00148	cardiac muscle adaptation	23	0.674345	1.49416	0.03825666	0.03825666	1	2900	tags=30%, Myh6/Gata6/Tcap/Nppa/Atp [illegible]
BP	GO:00032	muscle hypertrophy in response	22	0.685576	1.50554	0.03826547	0.03826547	1	2900	tags=32%, Myh6/Gata6/Tcap/Nppa/Atp [illegible]
BP	GO:00148	cardiac muscle hypertrophy in re	22	0.685576	1.50554	0.03826547	0.03826547	1	2900	tags=32%, Myh6/Gata6/Tcap/Nppa/Atp [illegible]
BP	GO:00069	hyperosmotic response	20	-0.65171	-1.51055	0.03863897	0.03863897	1	472	tags=25%, Efhd1/Slc12a2/Trpv4/Aqp1/ [illegible]
BP	GO:00327	positive regulation of interleukin	46	-0.52572	-1.43556	0.03999614	0.03999614	1	2234	tags=33%, Aim2/Tlr2/Tyrobp/Ccl19/Py [illegible]
BP	GO:00031	morphogenesis of an endotheliu	17	-0.69988	-1.56165	0.04001499	0.04001499	1	1596	tags=29%, Rbpj/Adamts12/Itgax/Alox1 [illegible]
BP	GO:00611	endothelial tube morphogenesis	17	-0.69988	-1.56165	0.04001499	0.04001499	1	1596	tags=29%, Rbpj/Adamts12/Itgax/Alox1 [illegible]
CC	GO:00992	intrinsic component of synaptic	239	0.428964	1.29432	0.04074074	0.04074074	1	5210	tags=37%, Scn11a/Glra4/Slc1a7/Slc6a5 [illegible]
BP	GO:00518	killing of cells in other organism	19	0.685204	1.46476	0.04091653	0.04091653	1	1785	tags=37%, Camp/Mbl2/F2rl1/Elane/F2/ [illegible]
BP	GO:00148	response to muscle activity	14	0.748633	1.49605	0.04139073	0.04139073	1	376	tags=14%, Myog/Prkag3
BP	GO:20001	positive regulation of cell motili	546	-0.31027	-1.1452	0.04139228	0.04139228	1	2896	tags=19%, Sdc4/Fgf10/Sema4d/Rras/Lg [illegible]
BP	GO:00108	positive regulation of T cell chem	11	-0.77294	-1.57893	0.04148728	0.04148728	1	1359	tags=36%, Adam17/Ccr2/Ccr7/Tmem10 [illegible]
BP	GO:20001	negative regulation of leukocyte	53	-0.49955	-1.411	0.04184663	0.04184663	1	2687	tags=28%, Il18/Nod2/Serpinb9/Blm/Cx [illegible]
BP	GO:20004	positive regulation of lymphocyt	32	-0.57962	-1.47515	0.04206403	0.04206403	1	3415	tags=34%, Selenok/Cxcl12/Itga4/Adam [illegible]
BP	GO:00518	killing by host of symbiont cells	16	0.718762	1.49134	0.04241436	0.04241436	1	1785	tags=44%, Camp/Mbl2/F2rl1/Elane/F2/ [illegible]
BP	GO:01201	tight junction assembly	58	-0.50348	-1.43586	0.04271193	0.04271193	1	2937	tags=34%, Nedd4l/Fzd5/Tbcd/Pecam1/ [illegible]
CC	GO:19904	omegasome	6	-0.8494	-1.55142	0.04290561	0.04290561	1	86	tags=33%, Emc6/Trim5
BP	GO:00019	positive regulation of cytokine-m	46	-0.52257	-1.42695	0.04302285	0.04302285	1	3237	tags=37%, Irf7/Adam17/Parp9/Mavs/La [illegible]
BP	GO:00301	vasopressin secretion	3	-0.93351	-1.45609	0.04386507	0.04386507	1	472	tags=67%, Trpv4/Ecrg4
BP	GO:00344	post-translational protein acetyla	3	-0.93322	-1.45564	0.04386507	0.04386507	1	886	tags=67%, Esco2/Dscc1
CC	GO:00360	endolysosome membrane	3	-0.93294	-1.4552	0.04386507	0.04386507	1	1122	tags=67%, Clec16a/Tasl
BP	GO:00380	Fc-gamma receptor signaling pa	11	-0.77007	-1.57307	0.04432395	0.04432395	1	1536	tags=55%, Rap1a/Fcgr2b/Ptprc/Fcer1g/ [illegible]
BP	GO:00905	regulation of membrane permeab	68	-0.47515	-1.39653	0.04478834	0.04478834	1	3591	tags=30%, Bnip3l/Naif1/Pdcd6ip/Mtch2 [illegible]
BP	GO:00026	positive regulation of germinal c	5	0.874253	1.44397	0.04482759	0.04482759	1	84	tags=20%, Tnfrsf13c
BP	GO:00189	germ-line sex determination	3	-0.93207	-1.45385	0.04520444	0.04520444	1	151	tags=67%, Sox9/Tmem184a
BP	GO:00095	detection of visible light	30	0.614908	1.43387	0.04538341	0.04538341	1	2163	tags=37%, Tulp1/Aipl1/Pde6c/Cacna1f [illegible]
BP	GO:00620	positive regulation of mitotic sis	3	0.932808	1.41417	0.0454029	0.0454029	1	770	tags=33%, Cdt1
CC	GO:00329	myosin filament	17	0.704643	1.48403	0.04552846	0.04552846	1	1672	tags=41%, Myh6/Myh4/Myh1/Myh8/M [illegible]
BP	GO:00018	complement activation, lectin pa	9	0.805086	1.47322	0.04591837	0.04591837	1	495	tags=33%, Colec11/Mbl2/Masp2
BP	GO:00300	muscle filament sliding	9	0.80489	1.47286	0.04591837	0.04591837	1	926	tags=33%, Myh6/Tnnt2/Myh8
CC	GO:00996	integral component of synaptic r	220	0.431693	1.29498	0.04648241	0.04648241	1	5210	tags=37%, Scn11a/Glra4/Slc1a7/Slc6a5 [illegible]
BP	GO:19001	positive regulation of renin secre	3	0.922626	1.39874	0.04701627	0.04701627	1	1366	tags=67%, F2rl1/Olfr78
CC	GO:00331	interphotoreceptor matrix	3	0.931155	1.41167	0.04757786	0.04757786	1	1282	tags=100% Rbp3/Impg2/Rtbdn
CC	GO:00451	intermediate filament cytoskelet	113	-0.42115	-1.30143	0.0477292	0.0477292	1	2408	tags=19%, Nefl/Bfsp2/Gfap/Cldn11/Stn [illegible]
CC	GO:00058	intermediate filament	75	-0.47221	-1.39704	0.04784513	0.04784513	1	2408	tags=24%, Nefl/Bfsp2/Gfap/Cldn11/Pcp [illegible]
MF	GO:00152	stretch-activated, cation-selectiv	3	-0.92825	-1.44789	0.04855285	0.04855285	1	472	tags=67%, Tmem28/Trpv4
BP	GO:00722	metanephric loop of Henle devel	3	-0.92785	-1.44726	0.04855285	0.04855285	1	176	tags=67%, Pou3f3/Aqp1
BP	GO:00507	regulation of viral process	148	-0.37249	-1.21562	0.04875869	0.04875869	1	2416	tags=21%, Triml1/Trim59/Trim30a/Ifitn [illegible]
MF	GO:00500	leukotriene-B4 20-monooxygena	4	0.896555	1.43105	0.04878049	0.04878049	1	779	tags=50%, Cyp4f18/Cyp4a31
BP	GO:00442	sulfur compound catabolic proce	19	-0.67019	-1.5282	0.04883387	0.04883387	1	2029	tags=26%, Chac1/Ggt1/Nudt7/Dpep1/M [illegible]
BP	GO:00095	detection of light stimulus	48	0.569664	1.42997	0.04885496	0.04885496	1	2163	tags=35%, Tulp1/Aipl1/Pde6c/Cacna1f [illegible]
BP	GO:00450	negative thymic T cell selection	13	-0.72812	-1.53242	0.04908494	0.04908494	1	2190	tags=38%, Zap70/Ptprc/Cd28/Ccr7/Aire [illegible]
BP	GO:00450	regulation of innate immune resp	163	-0.38553	-1.26866	0.04954535	0.04954535	1	2614	tags=26%, Lag3/Nod2/Lgals9/Polr3f/Ir [illegible]
BP	GO:00028	positive regulation of response t	135	-0.41023	-1.29685	0.04976977	0.04976977	1	2614	tags=36%, Nmi/Parp9/Mr1/Mavs/Srebf [illegible]
BP	GO:00027	regulation of antimicrobial humo	5	0.866652	1.43141	0.05	0.05	1	655	tags=40%, Gata6/Acod1
BP	GO:00506	dermatan sulfate proteoglycan bi	5	0.867282	1.43245	0.05	0.05	1	87	tags=20%, Chst14

r43/Wnt10b/Olfr466/Olfr628/Olfr1277/Scnn1a/Olfr107/Olfr1195/Olfr354/Olfr305/Olfr893/Olfr432/Lpo/Olfr740/Olfr204/Olfr1129/Olfr639/Olfr715/Olfr1356/Tas1r3/Olfr351/Olfr145/Olfr975/Olfr675/Olfr620/Ol
5/Olfr893/Olfr432/Olfr740/Olfr204/Olfr1116/Olfr1129/Olfr639/Olfr715/Olfr1356/Olfr351/Olfr145/Olfr975/Olfr675/Olfr620/Olfr1336/Olfr324/Olfr986/Olfr655/Olfr52/Olfr1048/Olfr90/Olfr129/Olfr
lfr432/Olfr740/Olfr204/Olfr1129/Olfr639/Olfr715/Olfr1356/Olfr351/Olfr145/Olfr975/Olfr675/Olfr620/Olfr1336/Olfr324/Olfr986/Olfr655/Olfr52/Olfr1048/Olfr90/Olfr129/Nav2/Olfr63/Olfr446/Olf
Olfr735/Olfr1209/Olfr671/Cnga2/Olfr279/Olfr510/Olfr125/Olfr853/Olfr1167/Vmn1r29/Olfr78/Opn1sw/Olfr286/Olfr774/Olfr904/Olfr613/Trpm5/Olfr905/Itpr3/Shank1/Olfr646/Olfr1247/Olfr127/Olfr398/Vmn1r43/Wnt10b/Pcdh15/Es

Supplement Table S2b. Gene set enrichment analysis of all expressed genes in the striatum

NTOLOG	ID	Description	setSize	richmentSc	NES	pvalue	p.adjust	qvalues	rank	ading_edge_enrichment
BP	GO:00516	defense response to virus	224	-0.47088	-1.71259	1.337E-06	1.337E-06	0.00982	2857	tags=27% Cgas/Unc93b1/F2rl1/Aim2/Ifitm7/Ticam2/Dhx58/Oas3/Zmynd11/Il27/Cd40/Ptpn22/Irf7/Il33/Parp9/Stat1/Pla2g10/Ifitm3/Cd86/Il15/Tagap/[illegible]
BP	GO:01405	defense response to symbiont	224	-0.47088	-1.71259	1.337E-06	1.337E-06	0.00982	2857	tags=27% Cgas/Unc93b1/F2rl1/Aim2/Ifitm7/Ticam2/Dhx58/Oas3/Zmynd11/Il27/Cd40/Ptpn22/Irf7/Il33/Parp9/Stat1/Pla2g10/Ifitm3/Cd86/Il15/Tagap/[illegible]
BP	GO:00096	response to virus	274	-0.43016	-1.58329	8.1617E-06	8.1617E-06	0.03995	2881	tags=25% Lgals9/Cgas/Unc93b1/F2rl1/Aim2/Ifitm7/Ticam2/Dhx58/Oas3/Zmynd11/Il27/Cd40/Ptpn22/Irf7/Il33/Parp9/Stat1/Pla2g10/Ifitm3/Cd86/Il15/[illegible]
BP	GO:00712	cellular response to molecule of bacterial	170	-0.46962	-1.64972	2.6275E-05	2.6275E-05	0.07873	2947	tags=23% Ccl12/Prdm1/Cd84/Cx3cr1/Cxcl1/Ticam2/Gbp2/Cxcl13/Pf4/Ptpn22/Cd274/Stat1/Cxcl16/Cd86/Hmgb1/Tnfrsf1b/Acod1/Trem2/Traip3/Casp1/[illegible]
BP	GO:00450	innate immune response	637	-0.33786	-1.32603	2.6806E-05	2.6806E-05	0.07873	3135	tags=24% Ifit2/Casp4/Ighd/Padi4/Ptk6/Ifi35/Mpeg1/Ccl12/Il18rap/Iglll1/Lgals9/Cgas/Prdm1/Unc93b1/Cd84/F2rl1/Gbp9/H2bc6/Cx3cr1/Aim2/Ifitm7/[illegible]
BP	GO:00712	cellular response to biotic stimulus	194	-0.43922	-1.55774	5.9173E-05	5.9173E-05	0.11926	2947	tags=22% Ccl12/Prdm1/Cd84/Cx3cr1/Cxcl1/Ticam2/Gbp2/Cxcl13/Pf4/Btk/Ptpn22/Cd274/Stat1/Cxcl16/Cd86/Hmgb1/Tnfrsf1b/Acod1/Trem2/Traip3/[illegible]
CC	GO:00985	side of membrane	591	-0.34054	-1.34621	6.2096E-05	6.2096E-05	0.11926	4226	tags=28% Gnao1/Capn2/Ermap/Syk/Abcg2/Fcgr3/Amot/Itgb2/Fgfr3/Rgs8/Atp2c2/Vcam1/Ada/Ptpn3/Itga4/Hck/Epb41/Myh9/Myh10/Cfh/Btla/Itga6/[illegible]
BP	GO:00354	response to interferon-beta	56	-0.63872	-1.91167	6.4969E-05	6.4969E-05	0.11926	2761	tags=59% Aim2/Ifitm7/Gbp2/Ifi213/Gm5431/Irgm1/9930111J21Rik1/Stat1/Ifitm3/Ifi47/Ikbke/Bst2/Tgtp1/Acod1/Gm4841/Igtp/F830016B08Rik/Gbp3/[illegible]
BP	GO:00022	activation of innate immune response	38	-0.70117	-1.97537	8.0786E-05	8.0786E-05	0.13182	2857	tags=37% Cgas/Aim2/Tyrobp/Zbp1/Tlr9/Nlrc4/Ifi204/Ifi209/Klrc1/Ifi203/Mndal/Ifi208/Klrk1/Ifi21
BP	GO:00712	cellular response to lipopolysaccharide	161	-0.46091	-1.6098	0.00010509	0.00010509	0.1431	2947	tags=23% Ccl12/Prdm1/Cd84/Cx3cr1/Cxcl1/Ticam2/Gbp2/Cxcl13/Pf4/Ptpn22/Cd274/Stat1/Cxcl16/Cd86/Hmgb1/Tnfrsf1b/Acod1/Trem2/Traip3/Casp1/[illegible]
BP	GO:00022	immune effector process	713	-0.3205	-1.2681	0.00011003	0.00011003	0.1431	2954	tags=21% Ifit2/Ripk3/Ighd/Enpp3/Prkcd/Ifi35/Ccr6/Il18rap/H2-K1/Nfkbid/Iglll1/Lgals9/Cgas/Unc93b1/Cd84/F2rl1/Cx3cr1/Aim2/Ifitm7/Ctsh/Trbc2/[illegible]
CC	GO:00098	external side of plasma membrane	404	-0.3569	-1.35912	0.00011694	0.00011694	0.1431	3795	tags=31% Cfh/Btla/Itga6/Ly6c1/Flt3/Il31ra/Car4/Ly6a/Ldlr/Thbd/Cd5/Fcer1g/Robo4/Ceacam1/Gp1ba/Clec14a/Cd200r3/L1cam/Ighm/Enpep/Nrg1/[illegible]
BP	GO:00300	myeloid cell differentiation	389	-0.36053	-1.36523	0.0001438	0.0001438	0.16244	5395	tags=33% Fli1/Creb1/Lbr/Mafb/Tlr2/Mturn/Ikzf1/Clec1b/Mfhas1/Hcls1/Inpp5d/Prkdc/Tnfrsf11a/Rbfox2/Bcl6/Tgfb1/Slc25a38/Ets1/Relb/Csf1r/Vegfa/[illegible]
BP	GO:00096	response to bacterium	565	-0.33725	-1.31392	0.00019043	0.00019043	0.19975	2947	tags=24% Ccl12/H2-K1/Ptger3/Cd79b/Iglll1/Lgals9/Prdm1/Cd84/F2rl1/Gbp9/Ms4a1/H2bc6/Cx3cr1/Trbc2/Herc6/Hmcn1/Cxcl1/Ticam2/Gbp2/Ptger2/[illegible]
MF	GO:00049	G protein-coupled receptor activity	534	-0.33554	-1.2905	0.00022591	0.00022591	0.22117	3763	tags=27% Olfr78/Gpr176/Pgr15l/Mas1/F2r/Vmn1r5/Nlrp6/Fshr/Vmn2r5/S1pr2/Qrfpr/Cnr2/Vmn2r102/Vmn2r52/Vmn2r60/Vmn1r236/Vmn2r1/Rxfp4/[illegible]
BP	GO:00028	regulation of response to biotic stimulus	283	-0.37982	-1.41731	0.00045035	0.00045035	0.38638	3014	tags=22% Ifi35/Il18rap/Lgals9/Cgas/Prdm1/Cd84/F2rl1/Aim2/Cxcl1/Hrg/Dhx58/Il27/Ptpn22/Irgm1/Otop1/Irf7/Parp9/Cd274/Stat1/Il15/Tyrobp/Zc3hav1/[illegible]
BP	GO:00354	cellular response to interferon-beta	45	-0.64023	-1.85226	0.00045301	0.00045301	0.38638	2761	tags=64% Aim2/Gbp2/Ifi213/Gm5431/Irgm1/9930111J21Rik1/Stat1/Ifi47/Tgtp1/Acod1/Gm4841/Igtp/F830016B08Rik/Gbp3/Gm12185/Ifi204/Ifi209/[illegible]
BP	GO:00072	phospholipase C-activating G protein-cou	91	-0.52418	-1.69279	0.00048595	0.00048595	0.38638	3653	tags=37% Galr2/P2ry10b/Kiss1/Gpr20/F2r/Fshr/Gna15/Gpr139/Gpr174/Cmklr1/C5ar1/Gpr157/Ptger3/Tgm2/F2rl1/C3ar1/Cx3cr1/Edn1/Plcb2/Oprm1/[illegible]
BP	GO:00033	cilium movement	162	0.542571	1.53951	0.00053034	0.00053034	0.38638	3943	tags=40% Cfap157/Dnah6/Tekt3/Drc7/Wt1/Jhy/Wfdc6a/Hoatz/Nme8/Cfap206/Spag16/Daw1/Ttll6/Tacr2/Dnali1/Tekt1/Dnaaf1/Cfap54/Dnah9/[illegible]
BP	GO:00507	regulation of immune response	627	-0.32134	-1.25268	0.00057237	0.00057237	0.38638	3216	tags=21% Ctla4/Cmklr1/C5ar1/Themis2/Ripk3/Nfam1/Ighd/Enpp3/Skint4/Ifi35/Il18rap/H2-K1/Cd79b/Nfkbid/Iglll1/Lgals9/Cgas/Cd84/F2rl1/C3ar1/[illegible]
BP	GO:00488	erythrocyte development	41	-0.64963	-1.86081	0.00059747	0.00059747	0.38638	3679	tags=41% Sox6/Ercc2/Slc11a2/Klf2/Epo/Rhag/Tal1/Rhd/Pla2g10/Adgrf5/Adgrf4/Klf1/Hba-a2/Hbb-b1/Hba-a1/Alas2/Slc4a
BP	GO:00702	pyroptosis	20	-0.77058	-1.87406	0.00063724	0.00063724	0.38638	3366	tags=50% Naip5/Casp4/Aim2/Zbp1/Casp1/Nlrc4/Gsdmc/Nlrp1b/Gzma/Naip6
CC	GO:00058	hemoglobin complex	7	-0.92407	-1.72172	0.00064755	0.00064755	0.38638	1479	tags=86% Hba-a2/Hbb-b1/Hba-a1/Hbb-bt/Hbb-y/Hbq1
MF	GO:00317	haptoglobin binding	7	-0.92407	-1.72172	0.00064755	0.00064755	0.38638	1479	tags=86% Hba-a2/Hbb-b1/Hba-a1/Hbb-bt/Hbb-y/Hbq1
CC	GO:00315	motile cilium	198	0.528291	1.52831	0.00065778	0.00065778	0.38638	3921	tags=34% Tekt3/Cst11/Cfap52/Drc7/Catsperg2/Nme8/Spag16/Rsph1/Slc25a31/Spata18/Tacr2/Dnali1/Dnah9/Spaca9/Adcy10/Ak7/Spag17/[illegible]
BP	GO:00456	negative regulation of megakaryocyte dif	16	-0.79314	-1.82175	0.00079858	0.00079858	0.41764	2471	tags=56% Pf4/H4c14/H4c2/H4c12/H4c3/H4c1/H4c4/H4c9/H4c1
BP	GO:00018	regulation of cytokine production	665	-0.31312	-1.23647	0.00082912	0.00082912	0.41764	4358	tags=26% Ghsr/Cd200/Fn1/Appl1/Irf5/Yy1/Dicer1/Il23a/Il7/Il17ra/Capn2/Ermap/Syk/Fcgr3/Polr3f/Hspb1/Cyba/Gpam/Ppara/Ripk1/Hdac1/Spon2/Pld4/[illegible]
BP	GO:00076	sensory perception of chemical stimulus	567	-0.32368	-1.25897	0.00083882	0.00083882	0.41764	3440	tags=29% Olfr750/Olfr1441/Olfr316/Olfr1437/Olfr329/Olfr1284/Olfr373/Olfr1118/Olfr173/Olfr506/Olfr705/Olfr1402/Olfr692/Olfr287/Olfr1090/[illegible]
BP	GO:00076	sensory perception of smell	523	-0.32421	-1.25501	0.00088518	0.00088518	0.41764	3440	tags=31% Olfr1033/Olfr64/Olfr658/Olfr814/Olfr1417/Olfr524/Olfr1313/Olfr1246/Olfr750/Olfr1441/Olfr316/Olfr1437/Olfr329/Olfr1284/Olfr373/[illegible]
BP	GO:00347	DNA replication-independent nucleosom	27	-0.70357	-1.82782	0.00090506	0.00090506	0.41764	1010	tags=30% H4c14/H4c2/H4c12/H4c3/H4c1/H4c4/H4c9/H4c1
BP	GO:00015	microtubule bundle formation	101	0.588546	1.5888	0.00090667	0.00090667	0.41764	4169	tags=39% Cfap157/Jhy/Hoatz/Rp1l1/Cfap206/Spag16/Rsph1/Prc1/Daw1/Ttll6/Dnaaf1/Ak7/Spag17/Dnajb13/Cfap65/Dnah8/Cfap44/Hydin/Rsph4a/[illegible]
BP	GO:00515	regulation of dopamine uptake involved i	6	0.944603	1.61291	0.00093853	0.00093853	0.41764	46	tags=17% Snca
BP	GO:00519	regulation of catecholamine uptake invol	6	0.944603	1.61291	0.00093853	0.00093853	0.41764	46	tags=17% Snca
MF	GO:00049	olfactory receptor activity	512	-0.31928	-1.23234	0.00108774	0.00108774	0.46981	3638	tags=31% Olfr1033/Olfr64/Olfr658/Olfr814/Vmn2r5/Olfr1417/Olfr524/Olfr1313/Olfr1246/Olfr750/Olfr1441/Olfr316/Olfr1437/Olfr329/Olfr1284/[illegible]
BP	GO:01405	granzyme-mediated programmed cell dea	6	-0.91778	-1.65808	0.0011488	0.0011488	0.482	250	tags=50% Bnip3/Gzmb/Gzma
BP	GO:00026	negative regulation of immune system pr	396	-0.33857	-1.2798	0.00136294	0.00136294	0.55597	4340	tags=25% Cd200/Appl1/Fer/Kitl/Samhd1/Adtrp/Hspb1/Cnot7/Gpam/Muc4/Ada/Mettl3/Ubash3a/Hc/Grn/Btla/Flt3/Adar/Ldlr/Nlrp6/Laptm5/Cnr2/[illegible]
BP	GO:00326	interferon-alpha production	32	-0.65708	-1.76535	0.0015231	0.0015231	0.58083	4756	tags=44% Havcr2/Azi2/Tlr4/Ddx58/Flt3/Ptpn22/Irf7/Stat1/Zc3hav1/Hmgb1/Tlr9/Ifih1/Tlr8/Gbp4
BP	GO:00065	spermine biosynthetic process	3	0.983505	1.49034	0.00154189	0.00154189	0.58083	362	tags=33% Amd2
BP	GO:00028	positive regulation of response to biotic s	148	-0.43193	-1.48015	0.00158394	0.00158394	0.58083	3014	tags=29% Ifi35/Il18rap/Lgals9/Cgas/F2rl1/Aim2/Cxcl1/Hrg/Dhx58/Irgm1/Irf7/Parp9/Cd274/Tyrobp/Zc3hav1/Zbp1/Ikbke/Hmgb1/Slamf6/Lag3/Tlr9/[illegible]
CC	GO:00617	inflammasome complex	18	-0.76939	-1.82752	0.00164656	0.00164656	0.58083	3607	tags=50% Nlrp6/Naip5/Casp4/Aim2/Casp1/Nlrc4/Nlrp1b/Naip6/Nlrp1a
CC	GO:00007	nucleosome	67	-0.53517	-1.64663	0.00168106	0.00168106	0.58083	2072	tags=33% H3c15/Tnp2/H3c1/H4c14/H3c11/H3c7/Prm1/H1f6/H2bc3/H4c2/H4c12/H4c3/H4c1/H4c4/H2ab3/H3c3/H4c9/H1f5/H3c4/H4c18/H2bc22/[illegible]
BP	GO:00722	metanephric glomerulus vasculature deve	8	0.907008	1.62776	0.00171017	0.00171017	0.58083	587	tags=38% Aqp1/Tcf21/Wt1
BP	GO:00450	negative regulation of viral genome repli	47	-0.608	-1.76267	0.00171284	0.00171284	0.58083	2952	tags=38% Parp10/Srpk1/Ifitm7/Oas3/Ifitm3/Zc3hav1/Bst2/Isg20/Oasl1/Oas1g/Isg15/Rsad2/Oasl2/Oas1a/Tnf/Oas2/Ccl5/Lt
BP	GO:00063	DNA replication-independent nucleosom	26	-0.71819	-1.81686	0.00177111	0.00177111	0.58083	2072	tags=31% H4c14/H4c2/H4c12/H4c3/H4c1/H4c4/H4c9/H4c1
BP	GO:00327	positive regulation of interleukin-1 produ	54	-0.57446	-1.70955	0.00177987	0.00177987	0.58083	3366	tags=35% Naip5/Casp4/Lgals9/Aim2/Tyrobp/Hmgb1/Ccl19/Nlrp10/Trim16/Casp1/Ccl3/Nlrc4/Ifi204/Tnf/Mndal/Tlr8/Lilra5/Ifi211/Nlrp1a
BP	GO:00063	nucleosome assembly	90	-0.49781	-1.60613	0.00193073	0.00193073	0.59214	2097	tags=27% H3c14/H2bc7/Padi4/Chaf1a/H3c15/H3c1/H2bc4/H4c14/H3c11/H3c7/H1f6/H2bc3/H4c2/H4c12/H4c3/H4c1/H4c4/H3c3/H4c9/H1f5/H3c4/[illegible]
BP	GO:00350	axoneme assembly	69	0.61632	1.60352	0.00193776	0.00193776	0.59214	4063	tags=46% Cfap157/Jhy/Hoatz/Rp1l1/Cfap206/Spag16/Rsph1/Daw1/Dnaaf1/Ak7/Spag17/Dnajb13/Cfap65/Dnah8/Cfap44/Hydin/Rsph4a/Cfap43/[illegible]
BP	GO:00063	DNA replication-dependent nucleosome	30	-0.65259	-1.73137	0.00201513	0.00201513	0.59214	2896	tags=47% Chaf1a/H3c15/H3c1/H4c14/H3c11/H4c2/H4c12/H4c3/H4c1/H4c4/H4c9/H3c4/H4c18/H3c
BP	GO:00347	DNA replication-dependent nucleosome	30	-0.65259	-1.73137	0.00201513	0.00201513	0.59214	2896	tags=47% Chaf1a/H3c15/H3c1/H4c14/H3c11/H4c2/H4c12/H4c3/H4c1/H4c4/H4c9/H3c4/H4c18/H3c
BP	GO:00071	adenylate cyclase-modulating G protein-	205	-0.38278	-1.37244	0.00201614	0.00201614	0.59214	4347	tags=26% Galr2/Nos1/Gnao1/Adm/Htr1a/Rxfp2/Adgrg1/Adgrg2/Gpr176/Akap12/Fshr/S1pr2/Cnr2/Akap13/Galr1/Gna15/Sstr4/Gpr101/Ptger3/Htr1f/[illegible]
BP	GO:00356	helper T cell diapedesis	3	-0.97588	-1.52311	0.00209951	0.00209951	0.60454	209	tags=67% Crk/Ccl5
BP	GO:00421	neutrophil activation	32	-0.64758	-1.73983	0.00224491	0.00224491	0.62717	4183	tags=59% Syk/Kmt2e/Itgb2/Dnase1/Myd88/Grn/Fcer1g/Prkcd/Il18rap/F2rl1/Pram1/Il15/[illegible]
BP	GO:00450	positive regulation of innate immune resp	111	-0.45419	-1.50667	0.00226717	0.00226717	0.62717	3014	tags=30% Ifi35/Il18rap/Lgals9/Cgas/Aim2/Irgm1/Irf7/Parp9/Tyrobp/Zbp1/Ikbke/Hmgb1/[illegible]
BP	GO:00362	granulocyte activation	38	-0.62978	-1.77425	0.00232452	0.00232452	0.62717	4183	tags=53% Syk/Kmt2e/Itgb2/Dnase1/Myd88/Grn/Fcer1g/Enpp3/Prkcd/Il18rap/F2rl1/Pram1/[illegible]
CC	GO:00318	haptoglobin-hemoglobin complex	8	-0.88133	-1.70164	0.00234896	0.00234896	0.62717	1479	tags=88% Hp/Hba-a2/Hbb-b1/Hba-a1/Hbb-bt/Hbb-y/Hbq1
CC	GO:00059	cilium	636	0.429925	1.30854	0.00241779	0.00241779	0.63228	4279	tags=25% Cfap157/Dnah6/Tekt3/Cst11/Cfap52/Drc7/Cdhr1/Ttll10/Stoml3/Prph/Rgr/Hoatz/[illegible]
BP	GO:19039	regulation of viral life cycle	141	-0.43253	-1.47374	0.00247327	0.00247327	0.63228	2952	tags=21% Parp10/Cav2/Srpk1/Ifitm7/Oas3/Ifitm3/Zc3hav1/Bst2/Trim10/Isg20/Trim30c/[illegible]
BP	GO:00018	positive regulation of cytokine production	415	-0.33274	-1.27444	0.00249727	0.00249727	0.63228	4340	tags=28% Cd200/Appl1/Irf5/Il23a/Il7/Il17ra/Syk/Fcgr3/Polr3f/Hspb1/Cyba/Ripk1/Hdac1/[illegible]
CC	GO:00021	zona pellucida receptor complex	13	-0.81275	-1.76884	0.00262875	0.00262875	0.65429	1696	tags=23% Hspa1b/Zpbp2/Zp3r
BP	GO:00456	regulation of myeloid cell differentiation	200	-0.38725	-1.37898	0.00279232	0.00279232	0.68342	5372	tags=34% Creb1/Mafb/Mturn/Ikzf1/Hcls1/Inpp5d/Prkdc/Rbfox2/Tgfb1/Ets1/Csf1r/Lmo2/[illegible]
MF	GO:00053	oxygen carrier activity	9	-0.86844	-1.73099	0.00288356	0.00288356	0.69418	2068	tags=67% Mb/Hbb-b1/Hba-a1/Hbb-bt/Hbb-y/Hbq1a
BP	GO:00615	myeloid cell development	80	-0.48482	-1.50777	0.00304838	0.00304838	0.72202	3335	tags=29% Slc11a2/Klf2/Epo/Rhag/Ep300/Tal1/Nbeal2/Rhd/Pla2g10/Tyrobp/Adgrf5/[illegible]
BP	GO:00018	cytokine production	745	-0.29693	-1.17077	0.00320707	0.00320707	0.73426	4358	tags=25% Ghsr/Cd200/Fn1/Appl1/Irf5/Yy1/Dicer1/Il23a/Il7/Il17ra/Capn2/Dbh/Ermap/Syk/[illegible]
BP	GO:00327	positive regulation of interleukin-1 beta p	47	-0.58662	-1.7007	0.00322169	0.00322169	0.73426	2295	tags=36% Naip5/Casp4/Aim2/Tyrobp/Hmgb1/Ccl19/Trim16/Casp1/Ccl3/Nlrc4/Ifi204/[illegible]
BP	GO:00326	regulation of interferon-alpha production	28	-0.67298	-1.74658	0.00325002	0.00325002	0.73426	4756	tags=46% Havcr2/Tlr4/Ddx58/Flt3/Ptpn22/Irf7/Stat1/Zc3hav1/Hmgb1/Tlr9/Ifih1/Tlr8/[illegible]
BP	GO:00022	pattern recognition receptor signaling pat	154	-0.41126	-1.42302	0.00341366	0.00341366	0.75954	5442	tags=36% Sarm1/Otud4/Tlr2/Epg5/Mfhas1/Lsm14a/Lrrc19/Pum2/Tnfaip3/Havcr2/Lyn/[illegible]
CC	GO:00448	DNA packaging complex	75	-0.50627	-1.57749	0.0037662	0.0037662	0.82547	2072	tags=29% H3c15/Tnp2/H3c1/H4c14/H3c11/H3c7/Prm1/H1f6/H2bc3/H4c2/H4c12/H4c3/[illegible]
BP	GO:00327	positive regulation of interferon-alpha pro	23	-0.69509	-1.72306	0.00387476	0.00387476	0.83678	4440	tags=48% Tlr4/Ddx58/Flt3/Ptpn22/Irf7/Stat1/Zc3hav1/Hmgb1/Tlr9/Ifih1/Tlr8
BP	GO:00485	negative regulation of viral process	85	-0.48195	-1.5344	0.00407712	0.00407712	0.86772	2952	tags=26% Parp10/Srpk1/Ifitm7/Oas3/Stat1/Ifitm3/Fbln1/Zc3hav1/Bst2/Trim10/Isg20/[illegible]
MF	GO:00426	T cell receptor binding	17	-0.73996	-1.75249	0.00416491	0.00416491	0.87374	2142	tags=53% H2-K1/H2-D1/H2-Q4/H2-Q7/H2-T23/Skint2/H2-Q1/H2-Q6/Eps8l1
CC	GO:00329	protein-DNA complex	162	-0.39863	-1.38808	0.00429975	0.00429975	0.88932	2885	tags=19% Kdm5a/St18/Pot1a/Sp1/H3c15/Tnp2/Esr2/Ep300/H3c1/H4c14/H3c11/H3c7/[illegible]
MF	GO:01401	molecular carrier activity	57	-0.52482	-1.57809	0.00466086	0.00466086	0.93853	2068	tags=12% Mb/Hbb-b1/Hba-a1/Hbb-bt/Kpna2/Hbb-y/Hbq1
BP	GO:00314	chromatin assembly	141	-0.42021	-1.43174	0.00467331	0.00467331	0.93853	3067	tags=18% Padi4/Parp10/Chaf1a/H3c15/H3c1/Hmgb1/H2bc4/H4c14/Apobec1/H3c11/[illegible]
BP	GO:00347	nucleosome organization	127	-0.43534	-1.45613	0.00477112	0.00477112	0.93853	3747	tags=22% Smarcc1/H2bc18/Fshr/H3c14/H2bc7/Padi4/Chaf1a/H3c15/Tnp2/H3c1/H2bc4/[illegible]
BP	GO:00106	regulation of mRNA stability involved in	3	-0.96402	-1.5046	0.0047933	0.0047933	0.93853	464	tags=67% Hnrnpm/Igf2bp1
BP	GO:00456	regulation of megakaryocyte differentiati	32	-0.62311	-1.67408	0.00489062	0.00489062	0.94498	2547	tags=34% Lox/Pf4/H4c14/L3mbtl1/H4c2/H4c12/H4c3/H4c1/H4c4/H4c9/H4c1
BP	GO:00063	chromatin assembly or disassembly	163	-0.39869	-1.39174	0.00507391	0.00507391	0.95339	3067	tags=16% Padi4/Parp10/Chaf1a/H3c15/Tal1/H3c1/Hmgb1/H2bc4/H4c14/Apobec1/[illegible]
BP	GO:00316	regulation of nervous system process	179	-0.38585	-1.36578	0.00512332	0.00512332	0.95339	5810	tags=39% Syne1/Stx1b/Wasf3/Reln/Grin2a/Rims1/Pawr/Grm1/Npff/Zfhx2/Il1a/Chrna4/[illegible]
BP	GO:00510	positive regulation of DNA-binding trans	252	-0.35402	-1.29697	0.00518952	0.00518952	0.95339	4622	tags=25% Cd36/Traf1/Ddr2/Taf12/Rnf41/Tlr4/Ikbkg/Neurod1/Cd200/Stk3/Fer/Mdfi/[illegible]
BP	GO:00326	interleukin-1 beta production	84	-0.47394	-1.50064	0.0051938	0.0051938	0.95339	2803	tags=30% Gbp5/Naip5/Casp4/F2rl1/Cx3cr1/Aim2/Igf1/Tyrobp/Ghrl/Hmgb1/Ccl19/[illegible]
BP	GO:00313	regulation of defense response	530	-0.31076	-1.20067	0.00537851	0.00537851	0.96907	4358	tags=27% Ghsr/Cd200/Appl1/Dicer1/Il23a/C2cd4b/Il17ra/Apobec3/Samhd1/Fcgr3/[illegible]
BP	GO:00019	cell killing	161	-0.39947	-1.39522	0.0054112	0.0054112	0.96907	2938	tags=30% Il18rap/H2-K1/Lgals9/F2rl1/Ctsh/Cxcl1/Cxcl13/Pf4/Emp2/Cd59a/Tyrobp/[illegible]
BP	GO:00603	interferon-gamma-mediated signaling pat	20	-0.70941	-1.72529	0.0056165	0.0056165	0.99371	3304	tags=40% Arg1/Irgm1/Otop1/Parp9/Stat1/Parp14/Irgm2/Nlrc5
BP	GO:00026	regulation of immune effector process	383	-0.32463	-1.21619	0.00582026	0.00582026	0.99947	2938	tags=21% Il18rap/H2-K1/Nfkbid/Lgals9/Cgas/Cd84/F2rl1/Aim2/Tnfsf13/Cxcl1/Was/[illegible]
CC	GO:00970	ciliary plasm	122	0.543906	1.49797	0.00584418	0.00584418	0.99947	3724	tags=28% Dnah6/Ttll10/Rp1l1/Nme8/Cfap206/Spag16/Dnali1/Dnah3/Dnaaf1/Dnah9/[illegible]
BP	GO:19047	regulation of chaperone-mediated autoph	4	0.946422	1.51303	0.00592433	0.00592433	0.99947	46	tags=25% Snca
BP	GO:00326	interleukin-1 production	102	-0.44945	-1.47137	0.00592642	0.00592642	0.99947	3499	tags=29% F2r/Ceacam1/Gbp5/Naip5/Casp4/Lgals9/F2rl1/Cx3cr1/Aim2/Igf1/Tyrobp/[illegible]
BP	GO:00507	regulation of viral process	159	-0.39696	-1.38318	0.00598932	0.00598932	0.99947	2952	tags=19% Parp10/Cav2/Srpk1/Ifitm7/Oas3/Stat1/Ifitm3/Fbln1/Zc3hav1/Bst2/Trim10/[illegible]
BP	GO:00022	myeloid cell homeostasis	180	-0.37936	-1.33568	0.00612554	0.00612554	0.99968	5339	tags=34% Mafb/Ikzf1/Ccr2/Mfhas1/Hcls1/Inpp5d/Prkdc/Mertk/Rbfox2/Bcl6/Slc25a38/[illegible]
MF	GO:00055	odorant binding	208	-0.36714	-1.3223	0.00612672	0.00612672	0.99968	3434	tags=33% Olfr1441/Olfr1437/Vmn1r236/Olfr173/Olfr506/Olfr1402/Olfr1090/Olfr967/[illegible]
BP	GO:00072	I-kappaB kinase/NF-kappaB signaling	211	-0.36772	-1.32551	0.00655515	0.00655515	1	4964	tags=29% Angpt1/Tnfaip3/Card11/Relb/Trim21/Ltbr/Abl1/Azi2/Cd36/Traf1/Tifa/Nfkbia/[illegible]
BP	GO:00722	metanephric glomerulus morphogenesis	3	0.969479	1.46909	0.00656531	0.00656531	1	485	tags=33% Tcf21
BP	GO:00722	metanephric glomerulus vasculature mor	3	0.969479	1.46909	0.00656531	0.00656531	1	485	tags=33% Tcf21
BP	GO:00722	metanephric glomerular capillary formati	3	0.969479	1.46909	0.00656531	0.00656531	1	485	tags=33% Tcf21
BP	GO:20004	regulation of peroxidase activity	3	0.969081	1.46849	0.00683466	0.00683466	1	46	tags=33% Snca
BP	GO:00380	Fc-gamma receptor signaling pathway	12	-0.78708	-1.67864	0.00729783	0.00729783	1	1693	tags=58% Appl1/Fcer1g/Ptprc/Myo1g/Fcgr2b/Clec4e/Oscar
MF	GO:00083	voltage-gated anion channel activity	15	0.770417	1.56639	0.00765072	0.00765072	1	1080	tags=13% Clcnka/Slc17a3
MF	GO:00703	aromatase activity	15	0.770685	1.56694	0.00765072	0.00765072	1	1919	tags=47% Cyp2b23/Cyp2b10/Cyp3a57/Cyp2c55/Cyp2b19/Cyp2d9/Cyp4a12b
CC	GO:00059	axoneme	121	0.54422	1.49955	0.00801282	0.00801282	1	3724	tags=29% Dnah6/Ttll10/Rp1l1/Nme8/Cfap206/Spag16/Dnali1/Dnah3/Dnaaf1/Dnah9/[illegible]
BP	GO:00603	regulation of response to interferon-gamm	15	-0.735	-1.64503	0.00818947	0.00818947	1	3304	tags=47% Arg1/Irgm1/Otop1/Parp9/Parp14/Irgm2/Nlrc5
BP	GO:00603	regulation of interferon-gamma-mediated	15	-0.735	-1.64503	0.00818947	0.00818947	1	3304	tags=47% Arg1/Irgm1/Otop1/Parp9/Parp14/Irgm2/Nlrc5
BP	GO:00311	negative regulation of microtubule polym	15	0.768014	1.56151	0.008341	0.008341	1	46	tags=7% Snca
BP	GO:00456	negative regulation of myeloid cell differ	86	-0.46867	-1.49017	0.00853584	0.00853584	1	3499	tags=24% Ceacam1/Cdc73/Cartpt/Itpkb/Runx1/Ubash3b/Pf4/Twist2/H4c14/Gpr68/[illegible]
BP	GO:00614	T follicular helper cell differentiation	6	0.900531	1.53766	0.00862756	0.00862756	1	874	tags=33% Il6/Il21
BP	GO:00356	helper T cell extravasation	4	-0.93409	-1.53683	0.00891511	0.00891511	1	1264	tags=50% Ccl2/Ccl5
CC	GO:00977	9+2 motile cilium	136	0.516228	1.43945	0.00902208	0.00902208	1	3921	tags=36% Tekt3/Cst11/Catsperg2/Nme8/Spag16/Rsph1/Spata18/Tacr2/Dnali1/Dnah9/[illegible]
BP	GO:00717	tumor necrosis factor superfamily cytokin	168	-0.3694	-1.29501	0.00986113	0.00986113	1	5362	tags=38% Il1a/Tlr2/Ifngr1/Ube2j1/Ccr2/Arhgef2/Ptafr/Akap8/Angpt1/Tnfaip3/Zbtb20/[illegible]
BP	GO:00326	regulation of tumor necrosis factor produ	160	-0.38262	-1.32983	0.00991662	0.00991662	1	5362	tags=39% Il1a/Tlr2/Ifngr1/Ube2j1/Ccr2/Arhgef2/Ptafr/Akap8/Angpt1/Tnfaip3/Zbtb20/[illegible]
BP	GO:00450	regulation of innate immune response	177	-0.38602	-1.3636	0.00995039	0.00995039	1	3014	tags=24% Ifi35/Il18rap/Lgals9/Cgas/Aim2/Dhx58/Irgm1/Otop1/Irf7/Parp9/Tyrobp/Zbp1/[illegible]
BP	GO:00346	response to prostaglandin	20	-0.68945	-1.67675	0.00995389	0.00995389	1	2696	tags=35% Acaca/Ptger2/Cyp27b1/Ccl19/Aanat/Akr1c18/P2ry4
BP	GO:19034	positive regulation of lactation	4	0.934272	1.49361	0.01021545	0.01021545	1	1025	tags=50% Prl/Prl7d1
BP	GO:00390	pronephric nephron development	3	-0.94775	-1.47921	0.01078669	0.01078669	1	182	tags=67% Ahi1/Hnf1b
CC	GO:00987	mitochondrial protein-containing comple	258	0.456682	1.34021	0.01089068	0.01089068	1	8802	tags=63% Tomm20l/Supv3l1/Immp2l/Mrps35/Wdr93/Timm10b/Polg/Mrpl49/Timm21/[illegible]
BP	GO:00427	regulation of sulfur metabolic process	11	0.814298	1.55137	0.0109149	0.0109149	1	46	tags=9% Snca
BP	GO:00380	Fc receptor signaling pathway	15	-0.7257	-1.62423	0.01100826	0.01100826	1	1693	tags=60% Nr4a3/Appl1/Fer/Fcer1g/Ptprc/Myo1g/Fcgr2b/Clec4e/Oscar
MF	GO:00190	GMP binding	4	0.932356	1.49054	0.0112565	0.0112565	1	28	tags=25% Gbp2b
MF	GO:00048	serine-type endopeptidase inhibitor activi	86	0.548199	1.45094	0.01134247	0.01134247	1	3338	tags=35% Serpina3m/Serpina3k/Serpina1b/Serpina3f/Serpina3c/Slpi/Serpina3n/Wfdc2/[illegible]
BP	GO:00072	neuropeptide signaling pathway	80	-0.44849	-1.39479	0.01138837	0.01138837	1	3318	tags=31% Rxfp4/Cartpt/Galr1/Sstr4/Tenm1/Npbwr1/Ltb4r2/Npb/Ptgdr2/Oprm1/Adcyap1/[illegible]
CC	GO:00306	phagocytic vesicle membrane	22	-0.6635	-1.64287	0.01140922	0.01140922	1	2932	tags=41% H2-K1/Hrg/H2-D1/Tap1/H2-Q7/B2m/H2-T23/[illegible]/Dmbt1
BP	GO:00065	polyamine metabolic process	19	0.751229	1.59674	0.01184684	0.01184684	1	595	tags=16% Ldc1/Amd2/Aoc1
BP	GO:00313	positive regulation of vesicle fusion	15	0.754498	1.53403	0.01190858	0.01190858	1	1682	tags=20% Snca/Cltrn/Anxa1
MF	GO:00198	oxygen binding	21	-0.66167	-1.62722	0.01192325	0.01192325	1	2068	tags=43% Mb/Hba-a2/Hbb-b1/Hba-a1/Alb/Hbb-bt/Hbb-y/Hbq1a/Cyp2f
BP	GO:00022	toll-like receptor signaling pathway	111	-0.41517	-1.37721	0.01245537	0.01245537	1	5442	tags=41% Sarm1/Otud4/Tlr2/Epg5/Mfhas1/Lrrc19/Tnfaip3/Havcr2/Lyn/Cd36/Nfkbia/Tlr4/[illegible]
BP	GO:00519	regulation of sensory perception of pain	53	-0.50495	-1.49404	0.01251788	0.01251788	1	5460	tags=49% Grm1/Npff/Zfhx2/Il1a/Ptafr/Slc9a1/Grk2/Ncam1/Tlr4/Nos1/Fabp5/Grm3/F2r/[illegible]
CC	GO:00328	plasma membrane bounded cell projectio	163	0.48429	1.37441	0.01279651	0.01279651	1	3724	tags=23% Dnah6/Ttll10/Rp1l1/Nme8/Cfap206/Spag16/Dnali1/Dnah3/Dnaaf1/Dnah9/[illegible]
BP	GO:00302	megakaryocyte differentiation	54	-0.50789	-1.51145	0.01299861	0.01299861	1	2885	tags=28% Sp1/Ep300/Tal1/Lox/Nbeal2/Pf4/H4c14/L3mbtl1/H4c2/H4c12/H4c3/H4c1/[illegible]
BP	GO:00622	positive regulation of pattern recognition	42	-0.56518	-1.62319	0.01303352	0.01303352	1	3449	tags=40% Slc15a4/Cd300ld3/Pik3ap1/Ifi35/F2rl1/Ticam2/Tlr5/Dhx58/Ptpn22/Zc3hav1/[illegible]
BP	GO:00427	defense response to bacterium	224	-0.34202	-1.24393	0.01339148	0.01339148	1	3372	tags=32% Ighm/Naip5/H2bc7/Defb36/Hp/C5ar1/Ighd/Prkcd/Mpeg1/H2-K1/Iglll1/Lgals9/[illegible]
BP	GO:00488	otolith development	7	0.87209	1.53654	0.01399483	0.01399483	1	238	tags=14% Oc90
BP	GO:00439	regulation of biological process involved	172	-0.37039	-1.30089	0.01403586	0.01403586	1	2952	tags=19% Parp10/Cav2/Srpk1/F2rl1/Ifitm7/Cxcl1/Oas3/Stat1/Ifitm3/Fbln1/Zc3hav1/Bst2/[illegible]
BP	GO:00507	positive regulation of immune response	474	-0.30539	-1.14734	0.01428473	0.01428473	1	3216	tags=22% Ctla4/Cmklr1/C5ar1/Themis2/Nfam1/Ighd/Skint4/Ifi35/Il18rap/H2-K1/Cd79b/[illegible]
BP	GO:19035	regulation of tumor necrosis factor superf	162	-0.38057	-1.32519	0.01449841	0.01449841	1	5362	tags=39% Il1a/Tlr2/Ifngr1/Ube2j1/Ccr2/Arhgef2/Ptafr/Akap8/Angpt1/Tnfaip3/Zbtb20/[illegible]
MF	GO:00017	2'-5'-oligoadenylate synthetase activity	6	-0.85768	-1.5495	0.01476045	0.01476045	1	2540	tags=83% Oas3/Oas1g/Oasl2/Oas1a/Oas2
BP	GO:00343	response to type I interferon	44	-0.53454	-1.54653	0.01549559	0.01549559	1	4448	tags=34% Stat2/Samhd1/Cnot7/Myd88/Mettl3/Adar/Ifitm7/Irf7/Stat1/Ifitm3/Zbp1/Ikbke/[illegible]
BP	GO:00022	myeloid leukocyte activation	214	-0.34774	-1.26442	0.01557047	0.01557047	1	4463	tags=29% Camk4/Myo18a/Tlr4/Cd200/Fer/Muc5b/C1qa/Syk/Kmt2e/Fcgr3/Itgb2/Dnase1/[illegible]
BP	GO:00313	regulation of vesicle fusion	29	0.665018	1.51493	0.01561954	0.01561954	1	1983	tags=14% Snca/Cltrn/Anxa1/Cplx3
CC	GO:00361	sperm flagellum	124	0.523154	1.44244	0.01563193	0.01563193	1	3921	tags=36% Tekt3/Cst11/Catsperg2/Nme8/Spag16/Rsph1/Spata18/Tacr2/Dnali1/Spaca9/[illegible]
CC	GO:00428	MHC class I peptide loading complex	15	-0.70463	-1.57708	0.01604047	0.01604047	1	2932	tags=53% H2-K1/H2-D1/H2-Q4/Tap1/H2-Q7/B2m/H2-Q1/H2-Q6
BP	GO:00321	negative regulation of response to extern	356	-0.32538	-1.20016	0.0161874	0.0161874	1	3904	tags=23% Cnot7/Ppara/Ada/Mettl3/Alox5/Hc/D130043K22Rik/Grn/Rora/Cpb2/Adar/Ldlr/[illegible]
MF	GO:19031	cuprous ion binding	7	0.863164	1.52081	0.01622404	0.01622404	1	46	tags=14% Snca
BP	GO:00027	MyD88-dependent toll-like receptor signa	19	-0.66374	-1.59287	0.0163584	0.0163584	1	4440	tags=42% Tlr4/Myd88/Tlr13/Tlr5/Irf7/Tlr9/Tlr1/Tlr8
BP	GO:00481	behavioral response to cocaine	20	0.709088	1.52504	0.01643671	0.01643671	1	46	tags=5% Snca
BP	GO:00326	negative regulation of interleukin-10 prod	17	-0.68865	-1.63096	0.01662067	0.01662067	1	2806	tags=35% Cd84/Cd274/Tyrobp/Fcgr2b/Pdcd1lg2/Epx
BP	GO:00450	regulation of viral genome replication	75	-0.45871	-1.4293	0.01677882	0.01677882	1	2952	tags=33% Dicer1/Apobec3/Cnot7/Ppara/Eif2ak2/Srpk2/Adar/Parp10/Srpk1/Ifitm7/Oas3/[illegible]
BP	GO:00650	protein-DNA complex assembly	162	-0.37746	-1.31434	0.01679512	0.01679512	1	2896	tags=19% Dlgap5/H2bc18/Cenpo/H3c14/H2bc7/Padi4/Chaf1a/H3c15/Ep300/Mcmdc2/[illegible]
BP	GO:00326	type I interferon production	92	-0.43595	-1.41445	0.01715597	0.01715597	1	4853	tags=51% Peli3/Traf3ip1/Crebbp/Cd14/Ddx3x/Ppm1b/Irf1/Polr3a/Tlr7/Dhx33/Flot1/Pola1/[illegible]
BP	GO:00326	tumor necrosis factor production	165	-0.3746	-1.30959	0.01723569	0.01723569	1	5362	tags=38% Il1a/Tlr2/Ifngr1/Ube2j1/Ccr2/Arhgef2/Ptafr/Akap8/Angpt1/Tnfaip3/Zbtb20/[illegible]
BP	GO:00721	positive regulation of mesenchymal to ep	4	-0.91844	-1.51108	0.01757035	0.01757035	1	1705	tags=75% Lif/Pax2/Pax8
BP	GO:00324	positive regulation of type I interferon pr	55	-0.4846	-1.44618	0.01766042	0.01766042	1	7219	tags=56% Cd14/Ddx3x/Irf1/Polr3a/Tlr7/Dhx33/Flot1/Ptpn11/Trim15/Chuk/Tlr2/Plcg2/[illegible]
CC	GO:00426	MHC protein complex	21	-0.644	-1.58376	0.01771759	0.01771759	1	2932	tags=52% H2-K1/H2-Ob/H2-D1/H2-Q4/H2-Q7/H2-M3/H2-Oa/B2m/H2-T23/H2-Q1/[illegible]
BP	GO:00488	homeostasis of number of cells	332	-0.33174	-1.24368	0.01773291	0.01773291	1	5426	tags=32% Akt3/Skil/Kras/Mafb/Ikzf1/Ccr2/Mfhas1/Lipa/Hcls1/Inpp5d/Prkdc/Lrrc19/[illegible]
MF	GO:00317	hemoglobin alpha binding	3	-0.93745	-1.46314	0.01803908	0.01803908	1	1420	tags=100% Hbb-bt/Hbb-y
MF	GO:00481	tau protein binding	22	0.703396	1.52909	0.01808318	0.01808318	1	46	tags=5% Snca
BP	GO:00326	regulation of interleukin-1 beta productio	74	-0.46287	-1.45466	0.01818432	0.01818432	1	2762	tags=26% Cx3cr1/Aim2/Igf1/Tyrobp/Ghrl/Hmgb1/Ccl19/Trem2/Trim16/Casp1/Ccl3/[illegible]
BP	GO:00354	SNARE complex assembly	20	0.705664	1.51767	0.01822571	0.01822571	1	927	tags=10% Snca/Cltrn
BP	GO:00354	response to interferon-alpha	24	-0.63592	-1.58297	0.01860904	0.01860904	1	3840	tags=42% Eif2ak2/Adar/Ifit2/Ifitm7/Ifitm3/Bst2/Tgtp1/Ifi204/Ifit3/Ifit
BP	GO:00327	negative regulation of interleukin-5 produ	4	-0.91335	-1.50272	0.0186141	0.0186141	1	9	tags=50% Lef1/Epx
MF	GO:00040	aryl sulfotransferase activity	3	0.950475	1.44029	0.01863297	0.01863297	1	540	tags=67% Sult1c2/Sult1c1
BP	GO:00315	activation of phospholipase D activity	6	-0.84821	-1.5324	0.0187466	0.0187466	1	209	tags=33% Arl1/Ccl5
MF	GO:00047	steroid delta-isomerase activity	4	0.921216	1.47274	0.01896251	0.01896251	1	1064	tags=50% Hsd3b6/Hsd3b3
MF	GO:00705	adenylyltransferase activity	31	-0.56534	-1.50595	0.01905062	0.01905062	1	1275	tags=19% Oas3/Oas1g/Tent5d/Oasl2/Oas1a/Oas2
MF	GO:00422	MHC class I protein binding	18	-0.6783	-1.61115	0.01914375	0.01914375	1	2932	tags=28% H2-K1/Tap1/H2-T23/Pilrb1/Klrk1
MF	GO:00422	MHC protein binding	28	-0.60105	-1.55991	0.01915105	0.01915105	1	2039	tags=25% H2-K1/Lag3/Tap1/H2-T23/Pilrb1/Col2a1/Klrk1
BP	GO:00519	sulfation	17	0.726811	1.50674	0.01937615	0.01937615	1	3957	tags=41% Sult1c2/Sult1c1/Sult6b1/Chst4/Sult6b2/Sult2b1/Sult5a1
BP	GO:00603	type I interferon signaling pathway	38	-0.55604	-1.56651	0.01940093	0.01940093	1	4448	tags=37% Stat2/Samhd1/Cnot7/Myd88/Mettl3/Adar/Ifitm7/Irf7/Stat1/Ifitm3/Zbp1/Ikbke/[illegible]
BP	GO:00713	cellular response to type I interferon	38	-0.55604	-1.56651	0.01940093	0.01940093	1	4448	tags=37% Stat2/Samhd1/Cnot7/Myd88/Mettl3/Adar/Ifitm7/Irf7/Stat1/Ifitm3/Zbp1/Ikbke/[illegible]
MF	GO:00051	prolactin receptor binding	4	0.920519	1.47162	0.0194856	0.0194856	1	1025	tags=50% Prl/Prl7d1
BP	GO:00519	regulation of sensory perception	54	-0.49671	-1.47819	0.01970374	0.01970374	1	5460	tags=48% Grm1/Npff/Zfhx2/Il1a/Ptafr/Slc9a1/Grk2/Ncam1/Tlr4/Nos1/Fabp5/Grm3/F2r/[illegible]
BP	GO:00607	positive regulation of response to cytokin	54	-0.49681	-1.47848	0.01970374	0.01970374	1	4440	tags=35% Tlr4/Il7/Ripk1/Ddx58/Laptm5/Casp4/Ticam2/Edn1/Irgm1/Irf7/Parp9/Zbp1/[illegible]
BP	GO:00106	negative regulation of platelet-derived gr	13	0.77108	1.52241	0.01972203	0.01972203	1	46	tags=8% Snca
MF	GO:00168	intramolecular oxidoreductase activity, tr	14	0.787795	1.57878	0.01989335	0.01989335	1	1064	tags=29% Idi2/Eci3/Hsd3b6/Hsd3b3
BP	GO:00313	regulation of cell killing	103	-0.41478	-1.35511	0.02010194	0.02010194	1	2938	tags=35% Il18rap/H2-K1/Lgals9/F2rl1/Cxcl1/Cd59a/Tyrobp/H2-T22/H2-D1/Slamf6/Lag3/[illegible]
MF	GO:00302	protein tyrosine kinase inhibitor activity	6	-0.84375	-1.52433	0.0202026	0.0202026	1	975	tags=50% Hyal2/Lilr4b/Lilrb4a
BP	GO:00324	regulation of type I interferon production	86	-0.43776	-1.39189	0.02061603	0.02061603	1	4853	tags=51% Peli3/Traf3ip1/Crebbp/Cd14/Ddx3x/Ppm1b/Irf1/Polr3a/Tlr7/Dhx33/Flot1/Pola1/[illegible]
BP	GO:00021	desmosome assembly	4	0.917515	1.46682	0.02079334	0.02079334	1	772	tags=25% Pkp3
BP	GO:00333	putrescine biosynthetic process from orni	4	0.918332	1.46812	0.02079334	0.02079334	1	135	tags=25% Ldc1
BP	GO:00026	negative regulation of leukocyte activatio	180	-0.35563	-1.25215	0.02081189	0.02081189	1	3824	tags=36% Pawr/Il4ra/Cd80/Ccr2/Gli3/Dlg5/Inpp5d/Dusp3/Lrrc32/Mertk/Bank1/Bcl6/[illegible]
BP	GO:00326	regulation of interleukin-1 production	90	-0.43656	-1.40849	0.02082014	0.02082014	1	2881	tags=29% F2r/Ceacam1/Naip5/Casp4/Lgals9/Cx3cr1/Aim2/Igf1/Tyrobp/Ghrl/Hmgb1/[illegible]
BP	GO:00065	polyamine biosynthetic process	14	0.785953	1.57509	0.02082402	0.02082402	1	362	tags=14% Ldc1/Amd2
BP	GO:00515	negative regulation of neurotransmitter tr	17	0.724479	1.5019	0.02102017	0.02102017	1	46	tags=6% Snca
MF	GO:00505	icosanoid binding	7	0.852124	1.50136	0.02117786	0.02117786	1	518	tags=29% Snca/S100a8
MF	GO:00505	arachidonic acid binding	7	0.852124	1.50136	0.02117786	0.02117786	1	518	tags=29% Snca/S100a8
BP	GO:00712	cellular response to copper ion	14	0.784115	1.5714	0.02152203	0.02152203	1	595	tags=21% Snca/Aqp1/Aoc1
BP	GO:00713	cellular response to nicotine	6	-0.83961	-1.51685	0.02165861	0.02165861	1	1445	tags=50% Ntrk1/B2m/Tnf
BP	GO:00329	positive regulation of superoxide anion g	23	-0.62448	-1.54803	0.02168152	0.02168152	1	4183	tags=52% Tgfb1/Syk/Itgb2/Cyba/Prkcd/F2rl1/Cxcl1/Crp/Tyrobp/Itgam/Cd177/Fpr2
BP	GO:00359	chondrocyte proliferation	20	-0.65885	-1.60234	0.02179432	0.02179432	1	3119	tags=40% Grn/Mmp16/Ccn3/Scube2/Ccn2/Comp/Six2/Ltf
MF	GO:00708	dynein complex binding	24	0.69474	1.53703	0.02220046	0.02220046	1	2727	tags=17% Snca/Dnaaf1/Ppp1r42/Kash5
BP	GO:00059	disaccharide metabolic process	6	-0.83652	-1.51126	0.02238661	0.02238661	1	2916	tags=67% Lalba/Fbp1/Fbp2/Treh
BP	GO:19046	cellular response to amyloid-beta	34	-0.56387	-1.53846	0.02267155	0.02267155	1	4713	tags=38% Gsk3b/Cd36/Tlr4/Vcam1/Itga4/Abcc1/Casp4/Ngfr/Igf1/Icam1/Trem2/Fpr2/[illegible]
BP	GO:00219	telencephalon regionalization	16	0.745662	1.54021	0.02354282	0.02354282	1	2978	tags=38% Eomes/Otx2/Emx1/Gsx2/Bmp4/Dmrta2
MF	GO:00505	icosatetraenoic acid binding	8	0.848236	1.52229	0.02389211	0.02389211	1	518	tags=25% Snca/S100a8
BP	GO:19015	regulation of endothelial tube morphogen	5	-0.88335	-1.50346	0.02417013	0.02417013	1	1953	tags=60% Itgax/Adamts12/Cxcl10
BP	GO:00508	regulation of acyl-CoA biosynthetic proce	5	0.897819	1.48711	0.02420116	0.02420116	1	46	tags=20% Snca
BP	GO:00346	response to prostaglandin E	17	-0.66952	-1.58565	0.02438819	0.02438819	1	2696	tags=47% Akap8/Ptger4/Acaca/Ptger2/Cyp27b1/Ccl19/Aanat/P2ry4
MF	GO:00430	alpha-tubulin binding	41	0.616074	1.47285	0.02465038	0.02465038	1	3534	tags=17% Snca/Cav3/1110017D15Rik/Trpv4/Dysf/Casc1/Efhc1
BP	GO:19027	positive regulation of chondrocyte prolife	8	-0.81382	-1.57129	0.02499636	0.02499636	1	925	tags=50% Scube2/Comp/Six2/Ltf
BP	GO:00972	hepatocyte apoptotic process	17	0.716654	1.48568	0.02525459	0.02525459	1	373	tags=12% Krt18/Krt8
BP	GO:00510	positive regulation of NF-kappaB transcri	144	-0.39048	-1.32615	0.02533304	0.02533304	1	3134	tags=17% Rps6ka5/Ripk3/Il18rap/Cd84/Cx3cr1/Aim2/Grem1/Lrrfip1/Cd40/Icam1/Tlr9/[illegible]
CC	GO:00058	axonemal dynein complex	21	0.69814	1.5054	0.02543298	0.02543298	1	3724	tags=52% Dnah6/Nme8/Dnah3/Dnah9/Dnah8/Dnah12/Cfap70/Dnhd1/Casc1/Dnah5/[illegible]
BP	GO:00508	regulation of liquid surface tension	3	0.943979	1.43045	0.02543322	0.02543322	1	1004	tags=67% Sftpd/Bpifa5
BP	GO:00604	positive regulation of gastric acid secretio	3	-0.92592	-1.44513	0.02565173	0.02565173	1	1376	tags=100% Trpv1/Kcnq1
BP	GO:00447	cilium organization	334	0.425514	1.2649	0.02587992	0.02587992	1	4224	tags=21% Cfap157/Tekt3/Cfap52/Noto/Jhy/Hoatz/Rp1l1/Nme8/Cfap206/Spag16/Rsph1/[illegible]
BP	GO:00027	regulation of leukocyte mediated immuni	224	-0.33144	-1.20545	0.02605395	0.02605395	1	2938	tags=23% Il18rap/H2-K1/Lgals9/Cd84/F2rl1/Tnfsf13/Cxcl1/Was/Pram1/Tbx21/Btk/Cd40/[illegible]
BP	GO:00508	regulation of cell activation	573	-0.2932	-1.15172	0.02623098	0.02623098	1	3124	tags=17% Zeb1/Ripk3/Nfam1/Ighd/Enpp3/Prkcd/Itpkb/Ptger3/Epo/Nfkbid/Iglll1/Lgals9/[illegible]
BP	GO:00302	erythrocyte differentiation	132	-0.38477	-1.29447	0.02640494	0.02640494	1	5339	tags=33% Mafb/Ikzf1/Mfhas1/Hcls1/Inpp5d/Prkdc/Rbfox2/Bcl6/Slc25a38/Ets1/Vegfa/[illegible]
BP	GO:00068	cellular copper ion homeostasis	16	-0.67979	-1.56141	0.02661095	0.02661095	1	483	tags=25% Atp7a/Atp7b/Prn/Prnd
CC	GO:19904	omegasome	6	-0.83194	-1.503	0.02675465	0.02675465	1	159	tags=33% Zfyve1/Trim5
BP	GO:00341	positive regulation of toll-like receptor si	29	-0.58353	-1.5249	0.02685927	0.02685927	1	3449	tags=48% Slc15a4/Cd300ld3/Pik3ap1/Ifi35/F2rl1/Ticam2/Tlr5/Ptpn22/Hmgb1/Tlr9/Tasl/[illegible]
BP	GO:00024	complement receptor mediated signaling	7	-0.82801	-1.54273	0.02687676	0.02687676	1	3201	tags=71% Cmklr1/C5ar1/C3ar1/Fpr2/C5ar2
BP	GO:19023	negative regulation of apoptotic process i	7	-0.82792	-1.54257	0.02687676	0.02687676	1	1734	tags=57% Foxc2/Pax2/Pax8/Hnf1b
CC	GO:00441	cytolytic granule	5	-0.8807	-1.49895	0.02699287	0.02699287	1	1101	tags=40% Prf1/Gzmb
BP	GO:00026	respiratory burst involved in defense resp	11	0.781306	1.48852	0.02722633	0.02722633	1	630	tags=18% Ins2/Lbp
MF	GO:00052	voltage-gated chloride channel activity	10	0.803097	1.50286	0.02762477	0.02762477	1	222	tags=10% Clcnka
CC	GO:00986	Schaffer collateral - CA1 synapse	112	-0.38402	-1.26668	0.02785518	0.02785518	1	8227	tags=53% Ppp3r1/Bsn/Nefh/Epha4/Stxbp5/Plcg1/Bcr/Rnf216/Rock2/Dgkb/Ptpra/Lrrtm2/[illegible]
BP	GO:00313	positive regulation of defense response	242	-0.3387	-1.2393	0.02790002	0.02790002	1	3014	tags=22% Ifi35/Il18rap/Ptger3/Tgm2/Lgals9/Cgas/F2rl1/Aim2/Cxcl1/Btk/Irgm1/Irf7/Il33/[illegible]
MF	GO:00048	complement receptor activity	7	-0.8252	-1.53749	0.02800443	0.02800443	1	3201	tags=71% Cmklr1/C5ar1/C3ar1/Fpr2/C5ar2
BP	GO:00301	natural killer cell activation	71	-0.44324	-1.37807	0.02812405	0.02812405	1	2881	tags=27% Lgals9/Prdm1/Il15ra/Ptpn22/Il15/Tyrobp/Slamf6/Ptprc/Gm13271/Fgr/H2-T23/[illegible]
MF	GO:00051	nerve growth factor receptor binding	4	0.902145	1.44225	0.02812443	0.02812443	1	1164	tags=50% Ntf5/Ntf3
BP	GO:00431	regulation of I-kappaB kinase/NF-kappaB	179	-0.35373	-1.2521	0.02847659	0.02847659	1	4964	tags=28% Angpt1/Tnfaip3/Card11/Trim21/Ltbr/Abl1/Cd36/Traf1/Tifa/Map3k14/Tlr4/[illegible]
BP	GO:00617	secretory granule maturation	4	-0.89721	-1.47615	0.02870374	0.02870374	1	919	tags=50% Snx19/Baiap3
CC	GO:00725	IPAF inflammasome complex	8	-0.80794	-1.55994	0.02906977	0.02906977	1	3366	tags=62% Naip5/Casp4/Casp1/Nlrc4/Naip6
BP	GO:00607	regulation of ribonuclease activity	12	-0.72847	-1.55365	0.02907923	0.02907923	1	2540	tags=50% Oas3/Oasl1/Oas1g/Oasl2/Oas1a/Oas2
BP	GO:00508	negative regulation of cell activation	201	-0.34774	-1.24279	0.02924739	0.02924739	1	3824	tags=23% Grn/Btla/Flt3/Ldlr/Laptm5/Cnr2/Ceacam1/Arg1/Ctla4/Enpp3/Prkcd/Ptger3/[illegible]
BP	GO:19905	dense core granule maturation	3	-0.9243	-1.44261	0.02929257	0.02929257	1	919	tags=67% Snx19/Baiap3
BP	GO:00019	leukocyte mediated cytotoxicity	118	-0.39561	-1.32008	0.02972625	0.02972625	1	2938	tags=32% Il18rap/H2-K1/Lgals9/F2rl1/Ctsh/Cxcl1/Emp2/Tyrobp/H2-T22/H2-D1/Slamf6/[illegible]
BP	GO:00140	positive regulation of phosphatidylinosito	75	-0.4357	-1.3576	0.02987927	0.02987927	1	4551	tags=36% Ube3a/Sema4d/Flt1/Pdgfb/Fn1/Myoc/Cbl/Nkx3-1/Tek/F2r/Fshr/Pik3ap1/Nrg1/[illegible]
MF	GO:00718	neuropeptide receptor binding	32	-0.5504	-1.47874	0.02997289	0.02997289	1	2948	tags=27% Apln/a/Edn1/Adcyap1/Crh/Spx/Pmch/Ucn3/Hcrt
BP	GO:00550	copper ion homeostasis	17	-0.65789	-1.55811	0.03012646	0.03012646	1	483	tags=24% Atp7a/Atp7b/Prn/Prnd
BP	GO:00336	positive regulation of osteoblast prolifera	14	-0.70202	-1.55076	0.03021324	0.03021324	1	19	tags=29% Gsk3b/Abl1/Hpse/Ltf
CC	GO:00989	GABA-ergic synapse	104	-0.40183	-1.326	0.03059287	0.03059287	1	7896	tags=57% Atp2b3/Drd1/Gabra4/Lrrtm2/Slitrk1/Nlgn3/Phb/Septin11/Gabbr2/Erbb4/[illegible]
BP	GO:00602	regulation of respiratory burst involved in	6	0.860474	1.46926	0.03078169	0.03078169	1	630	tags=33% Ins2/Lbp
BP	GO:00725	reactive oxygen species metabolic proces	280	-0.31567	-1.17463	0.03086292	0.03086292	1	3289	tags=18% Klf2/Hp/Klf4/Ripk3/Ngfr/Map3k7/Prkcd/Slc30a10/F2rl1/Esr2/Cx3cr1/Igf1/[illegible]
BP	GO:00509	detection of light stimulus involved in vis	22	-0.61757	-1.52916	0.03094565	0.03094565	1	3898	tags=45% Tulp1/Atp8a2/Best1/Rgs9bp/Sema5b/Cacnb4/Gucy2f/Gnat1/Cacna1f/Gja10
BP	GO:00509	detection of light stimulus involved in sen	22	-0.61757	-1.52916	0.03094565	0.03094565	1	3898	tags=45% Tulp1/Atp8a2/Best1/Rgs9bp/Sema5b/Cacnb4/Gucy2f/Gnat1/Cacna1f/Gja10
BP	GO:00357	ureter maturation	4	-0.89457	-1.47181	0.03113918	0.03113918	1	1800	tags=75% Aldh1a2/Gata3/Pax2
BP	GO:00351	embryonic hemopoiesis	29	-0.57631	-1.50603	0.03137753	0.03137753	1	6645	tags=55% Sh2b3/Med1/Tgfbr2/Stk4/Kmt2a/Vegfa/Lmo2/Atf4/Stk3/Kit/Kdr/Runx1/Tal1/[illegible]
BP	GO:00093	oligosaccharide metabolic process	38	-0.53814	-1.51607	0.03156627	0.03156627	1	4837	tags=39% B3galt2/Ctbs/B3galt1/St3gal6/St8sia2/St6gal2/St6galnac3/Hexb/Lalba/Fut4/[illegible]
BP	GO:00320	ISG15-protein conjugation	5	-0.87262	-1.4852	0.03193267	0.03193267	1	2192	tags=60% Uba7/Ube2l6/Isg15
BP	GO:00215	vestibulocochlear nerve development	9	-0.79107	-1.57677	0.03216748	0.03216748	1	1638	tags=44% Tifab/Pax2/Atp8b1/Pou4f3
BP	GO:00617	protein lipidation involved in autophagos	4	-0.89287	-1.46901	0.03218294	0.03218294	1	563	tags=50% Wipi2/Irgm2
BP	GO:00094	putrescine metabolic process	8	0.83183	1.49285	0.03243266	0.03243266	1	595	tags=25% Ldc1/Aoc1
BP	GO:00327	negative regulation of ATPase activity	18	-0.65064	-1.54545	0.03245796	0.03245796	1	19	tags=11% Tsc1/Ltf
BP	GO:00326	interleukin-10 production	53	-0.46688	-1.3814	0.03261466	0.03261466	1	3020	tags=28% Prkcd/Lgals9/Cd84/F2rl1/Cd34/Cd274/Tyrobp/Tlr9/Trem2/Isg15/Fcgr2b/Pdcd1/[illegible]
BP	GO:00109	negative regulation of endopeptidase acti	146	0.480822	1.34899	0.03271028	0.03271028	1	1564	tags=14% Serpina3m/Snca/Serpina3k/Serpina1b/Serpina3f/Serpina3c/Fetub/Aqp1/Il6/[illegible]
BP	GO:00720	mesenchymal stem cell maintenance invo	7	-0.81726	-1.5227	0.03289104	0.03289104	1	1638	tags=57% Pax2/Six2/Pax8/Hnf1b
MF	GO:00302	guanylate cyclase regulator activity	3	-0.92053	-1.43673	0.03293342	0.03293342	1	1318	tags=100% Pdzd3/Guca1b
BP	GO:00070	microtubule-based movement	361	0.415314	1.23766	0.03305785	0.03305785	1	3943	tags=21% Cfap157/Dnah6/Tekt3/Drc7/Wt1/Jhy/Wfdc6a/Hoatz/Nme8/Kif12/Cfap206/[illegible]
BP	GO:00190	viral life cycle	219	-0.33874	-1.22806	0.03312591	0.03312591	1	2952	tags=15% Parp10/Cav2/Srpk1/Ifitm7/Spint1/Oas3/Ifitm3/Icam1/Zc3hav1/Bst2/Trim10/[illegible]
BP	GO:19010	negative regulation of autophagosome ma	5	-0.87133	-1.483	0.03334405	0.03334405	1	1850	tags=40% Ubqln4/Clec16a
BP	GO:00720	DCT cell differentiation	3	-0.91977	-1.43554	0.03359539	0.03359539	1	1638	tags=67% Pax2/Pax8
BP	GO:00722	metanephric DCT cell differentiation	3	-0.91977	-1.43554	0.03359539	0.03359539	1	1638	tags=67% Pax2/Pax8
CC	GO:19905	perinuclear endoplasmic reticulum memb	3	0.934861	1.41663	0.03362303	0.03362303	1	300	tags=33% Mogat2
BP	GO:00718	protein-DNA complex subunit organizati	203	-0.34266	-1.23188	0.03371592	0.03371592	1	3747	tags=16% Smarcc1/Dlgap5/H2bc18/Fshr/Cenpo/H3c14/H2bc7/Padi4/Chaf1a/H3c15/Tnp2/[illegible]
BP	GO:00026	negative regulation of leukocyte migratio	40	-0.514	-1.46522	0.03395165	0.03395165	1	4340	tags=35% Cd200/Adtrp/Ada/Hc/Lrch1/Ccn3/Ccl12/Grem1/Il33/Mmp28/Gpr18/Dpp4/[illegible]
BP	GO:00355	regulation of SNARE complex assembly	9	0.82395	1.51391	0.03431555	0.03431555	1	927	tags=22% Snca/Cltrn
BP	GO:00313	positive regulation of cell killing	73	-0.4502	-1.40876	0.03431566	0.03431566	1	2938	tags=42% Il18rap/H2-K1/F2rl1/Cxcl1/Tyrobp/H2-T22/H2-D1/Slamf6/Lag3/Itgam/H2-Q4/[illegible]
BP	GO:00713	cellular response to cytokine stimulus	681	-0.27592	-1.08893	0.03456197	0.03456197	1	2978	tags=16% Csf2rb2/Adam23/Ccl12/Il18rap/Epo/Spp1/F2rl1/Gbp9/Cx3cr1/Aim2/Ifitm7/[illegible]
BP	GO:00189	germ-line sex determination	3	0.933903	1.41518	0.03471644	0.03471644	1	479	tags=33% Tmem184a
CC	GO:00360	endolysosome	7	-0.80955	-1.50833	0.03514641	0.03514641	1	3449	tags=86% Slc15a4/Prkcd/Hrg/Tlr9/Clec16a/Tasl
BP	GO:00026	regulation of leukocyte activation	529	-0.28498	-1.10527	0.03558067	0.03558067	1	3583	tags=19% Laptm5/Kmt5b/Cnr2/Trp53bp1/Cd5/Fcer1g/Ceacam1/Slc15a4/Ighm/Arg1/[illegible]
BP	GO:00104	negative regulation of peptidase activity	195	0.458172	1.32292	0.03571429	0.03571429	1	3505	tags=24% Serpina3m/Snca/Serpina3k/Serpina1b/Serpina3f/Serpina3c/Fetub/Aqp1/Cst11/[illegible]
MF	GO:00381	pattern recognition receptor activity	20	-0.62763	-1.52641	0.03616636	0.03616636	1	1973	tags=35% Tlr9/Clec7a/Pglyrp1/Dmbt1/Tlr8/Clec4e/Trim5
BP	GO:00190	viral genome replication	108	-0.38864	-1.28909	0.03617258	0.03617258	1	2952	tags=25% Dicer1/Apobec3/Cnot7/Ppara/Eif2ak2/Srpk2/Adar/Ceacam1/Parp10/Srpk1/[illegible]
BP	GO:20012	regulation of vasculogenesis	16	-0.6688	-1.53617	0.03624938	0.03624938	1	5592	tags=69% Rapgef2/Ramp2/Rin2/Adm/Asb4/Ceacam1/Kdr/Cd34/Tmem100/Emp2/Xdh
MF	GO:00037	double-stranded RNA binding	80	-0.41773	-1.29913	0.03628754	0.03628754	1	4938	tags=29% Hsp90ab1/Adarb1/Ago3/Mbnl1/Dicer1/Mdh/Eif2ak2/Ddx58/Adar/Hspa1a/[illegible]
CC	GO:00058	microtubule associated complex	140	0.475268	1.32456	0.03659976	0.03659976	1	3724	tags=21% Dnah6/Aurkc/Actl7b/Nme8/Kif12/Trim54/Dnali1/Dynlrb2/Dnah3/Dnah9/[illegible]
BP	GO:00071	adenylate cyclase-activating G protein-co	131	-0.37825	-1.27178	0.03671175	0.03671175	1	4932	tags=29% Cnr1/Adgrl2/Ramp2/Gnal/Ptger4/Grk2/Nos1/Adm/Rxfp2/Adgrg1/Adgrg2/Fshr/[illegible]
BP	GO:19035	negative regulation of ATP metabolic pro	31	-0.53309	-1.42003	0.03676798	0.03676798	1	2024	tags=19% Entpd1/Fbp1/Apoc3/Tnf/Slc4a1/Pde2a
BP	GO:00314	slow-twitch skeletal muscle fiber contract	3	-0.91698	-1.43118	0.03692156	0.03692156	1	1340	tags=100% Tnnt1/Strit1
BP	GO:19032	negative regulation of leukocyte tethering	3	-0.91666	-1.43068	0.03692156	0.03692156	1	111	tags=67% Cxcl12/Ccl28
BP	GO:00019	lymphangiogenesis	21	-0.60881	-1.49722	0.03695392	0.03695392	1	5483	tags=62% Ccbe1/Flt4/Vegfc/Vegfa/Pkd1/Epha2/Clec14a/Sox18/Tie1/Acvr2b/Foxc2/[illegible]
BP	GO:00616	glycolytic process through fructose-6-pho	14	-0.69236	-1.52943	0.03771435	0.03771435	1	114	tags=21% Galc/Foxk2/Slc4a1
MF	GO:00380	collagen receptor activity	7	-0.80735	-1.50425	0.03777766	0.03777766	1	1438	tags=43% Ddr2/Musk/Oscar
BP	GO:00068	xenobiotic metabolic process	65	0.555413	1.43449	0.03785901	0.03785901	1	1914	tags=20% Ugt1a6b/Cyp2b23/Nr1i2/Sult1c1/Cyp2j11/Fmo2/Slco1a6/Cyp2b10/Cyp2c55/[illegible]
MF	GO:00151	sialic acid transmembrane transporter acti	4	0.893173	1.4279	0.03811229	0.03811229	1	1080	tags=50% Slc17a1/Slc17a3
BP	GO:00157	sialic acid transport	4	0.893173	1.4279	0.03811229	0.03811229	1	1080	tags=50% Slc17a1/Slc17a3
CC	GO:00302	dynein complex	49	0.577695	1.42025	0.0384106	0.0384106	1	3724	tags=29% Dnah6/Nme8/Dnali1/Dynlrb2/Dnah3/Dnah9/Dynlt1b/Dnah8/Dnah12/Cfap70/[illegible]
BP	GO:00714	cellular response to xenobiotic stimulus	69	0.548809	1.42788	0.03870968	0.03870968	1	1914	tags=19% Ugt1a6b/Cyp2b23/Nr1i2/Sult1c1/Cyp2j11/Fmo2/Slco1a6/Cyp2b10/Cyp2c55/[illegible]
BP	GO:00358	negative regulation of urine volume	10	-0.74846	-1.53791	0.03885145	0.03885145	1	1050	tags=30% Avpr2/Slc5a2/Slc4a1
BP	GO:00602	cilium movement involved in cell motility	111	0.498403	1.36215	0.03956835	0.03956835	1	4672	tags=41% Cfap157/Tekt3/Drc7/Wt1/Wfdc6a/Hoatz/Nme8/Cfap206/Spag16/Tacr2/Tekt1/[illegible]
BP	GO:00424	indole-containing compound biosynthetic	8	-0.7932	-1.53148	0.03981974	0.03981974	1	1339	tags=50% Gch1/Aanat/Tph1/Fev
BP	GO:00447	multi-organism transport	12	-0.70826	-1.51055	0.03990859	0.03990859	1	6137	tags=75% Kpna6/Siglec1/Trim15/Pikfyve/Eps15/Tpcn2/Cav2/Bst2/Kpna2
BP	GO:00467	transport of virus	12	-0.70826	-1.51055	0.03990859	0.03990859	1	6137	tags=75% Kpna6/Siglec1/Trim15/Pikfyve/Eps15/Tpcn2/Cav2/Bst2/Kpna2
BP	GO:19025	multi-organism localization	12	-0.70826	-1.51055	0.03990859	0.03990859	1	6137	tags=75% Kpna6/Siglec1/Trim15/Pikfyve/Eps15/Tpcn2/Cav2/Bst2/Kpna2
BP	GO:00974	protein maturation by iron-sulfur cluster t	14	0.758134	1.51934	0.03998317	0.03998317	1	69	tags=7% AK157302
MF	GO:00045	beta-glucuronidase activity	3	0.928402	1.40684	0.04018349	0.04018349	1	704	tags=33% Kl
BP	GO:19037	regulation of hemopoiesis	360	-0.30964	-1.15628	0.04048274	0.04048274	1	5432	tags=31% Il4ra/Cd46/Creb1/Mafb/Atp11c/Mturn/Ikzf1/Ccr2/Gli3/Hcls1/Inpp5d/Prkdc/[illegible]
BP	GO:00713	cellular response to prostaglandin stimulu	15	-0.65981	-1.47675	0.04066785	0.04066785	1	2696	tags=40% Akap8/Ptger4/Acaca/Ptger2/Akr1c18/P2ry4
BP	GO:00350	negative regulation of histone acetylation	17	0.709111	1.47005	0.04081633	0.04081633	1	4801	tags=35% Snca/Mex3/Atg7/Brca1/Foxp3/Zfp451
MF	GO:00426	CD8 receptor binding	11	-0.73839	-1.53931	0.04096773	0.04096773	1	2932	tags=64% H2-K1/H2-D1/H2-Q4/H2-Q7/H2-M3/H2-Q1/H2-Q6
CC	GO:00426	MHC class I protein complex	11	-0.73483	-1.5319	0.04096773	0.04096773	1	2932	tags=73% H2-K1/H2-D1/H2-Q4/H2-Q7/B2m/H2-T23/H2-Q1/H2-Q6
BP	GO:00424	serotonin metabolic process	15	-0.6588	-1.47449	0.04108928	0.04108928	1	5600	tags=53% Grin2a/Cyp2d22/Btbd9/Gch1/Atp7a/Htr1a/Tph1/Fev
BP	GO:19011	primary amino compound metabolic proc	17	-0.63944	-1.51441	0.04119318	0.04119318	1	5600	tags=53% Grin2a/Cyp2d22/Chka/Btbd9/Gch1/Atp7a/Htr1a/Tph1/Fev
MF	GO:00085	ammonium transmembrane transporter ac	9	0.813688	1.49506	0.04122792	0.04122792	1	1406	tags=40% Slc12a6/Aqp1/Rhbg/Rhcg
BP	GO:00156	ammonium transport	9	0.813688	1.49506	0.04122792	0.04122792	1	1406	tags=40% Slc12a6/Aqp1/Rhbg/Rhcg
CC	GO:00356	sperm fibrous sheath	12	0.762207	1.4685	0.04133545	0.04133545	1	3611	tags=50% Nme8/Aldoart1/Akap14/Flacc1/Gapdhs/Akap3
BP	GO:00436	engulfment of apoptotic cell	18	-0.6342	-1.5064	0.04148588	0.04148588	1	4174	tags=67% Adgrb1/Xkr8/Abca7/Rhobtb1/Megf10/Rhoh/Rhobtb2/Xkr7/Xkr4/Alox15/[illegible]
BP	GO:00019	T cell mediated cytotoxicity	53	-0.4565	-1.35068	0.04159095	0.04159095	1	3116	tags=42% Ripk3/H2-K1/Ctsh/Emp2/H2-T22/H2-D1/H2-Q4/Hspa8/Gm11127/H2-Q7/[illegible]
CC	GO:00425	specific granule	13	-0.6919	-1.50582	0.04164961	0.04164961	1	1332	tags=23% Anxa3/Camp/Ltf
MF	GO:00198	isoprenoid binding	28	0.642921	1.45411	0.04184704	0.04184704	1	5077	tags=43% Lrat/Rbp2/Rho/Rbp3/Rbp7/Stra6/Opn4/Olfr49/Crabp1/Ptgds/Rara/Adh7
BP	GO:00514	response to corticosterone	6	0.846233	1.44494	0.04230118	0.04230118	1	1064	tags=50% Hsd3b6/Gm4450/Hsd3b3
BP	GO:20012	positive regulation of vasculogenesis	11	-0.73429	-1.53076	0.04255866	0.04255866	1	5592	tags=82% Rapgef2/Ramp2/Rin2/Adm/Asb4/Ceacam1/Kdr/Cd34/Tmem100
CC	GO:00331	proton-transporting V-type ATPase, V0 d	9	0.80638	1.48163	0.04269294	0.04269294	1	2831	tags=33% Atp6v0d2/Atp6v0a4/Atp6v0a2
BP	GO:00020	norepinephrine-epinephrine-mediated va	3	-0.9126	-1.42435	0.04290869	0.04290869	1	713	tags=100% Adrb1/Adrb3
BP	GO:00023	gamma-delta intraepithelial T cell differe	3	-0.90999	-1.42028	0.04290869	0.04290869	1	1828	tags=67% Gpr18/Klrc1
BP	GO:00023	CD8-positive, gamma-delta intraepithelia	3	-0.90999	-1.42028	0.04290869	0.04290869	1	1828	tags=67% Gpr18/Klrc1
MF	GO:00049	beta-adrenergic receptor activity	3	-0.9126	-1.42435	0.04290869	0.04290869	1	713	tags=100% Adrb1/Adrb3
CC	GO:00725	NLRP1 inflammasome complex	3	-0.90954	-1.41957	0.04290869	0.04290869	1	1702	tags=100% Casp1/Nlrp1b
BP	GO:00444	motile cilium assembly	26	0.655879	1.4716	0.04322767	0.04322767	1	4465	tags=46% Noto/Jhy/Dnaaf1/Cfap43/Dnaaf3/Zmynd10/Rsph6a/Cfap221/Ulk4/Foxj1/[illegible]
MF	GO:00228	wide pore channel activity	28	-0.5589	-1.4505	0.04333284	0.04333284	1	4024	tags=36% Gje1/Gja3/Gja5/Gjb6/Gja6/Gjc1/Gja8/Gjd4/Gja10/Prf1
MF	GO:00046	peroxidase activity	47	-0.4923	-1.42726	0.04356046	0.04356046	1	2376	tags=23% Iyd/Ptgs1/Lpo/Gpx6/Hba-a2/Hbb-b1/Hba-a1/Hbb-bt/Hbb-y/Hbq1a/Epx
BP	GO:00353	thioester biosynthetic process	25	0.652407	1.45166	0.04373178	0.04373178	1	46	tags=4% Snca
BP	GO:00716	acyl-CoA biosynthetic process	25	0.652407	1.45166	0.04373178	0.04373178	1	46	tags=4% Snca
BP	GO:00025	regulation of antigen processing and pres	5	-0.85905	-1.46211	0.04432395	0.04432395	1	2695	tags=60% H2-Ob/Trem2/H2-Oa
BP	GO:00322	negative regulation of synaptic transmissi	4	-0.87746	-1.44366	0.04440475	0.04440475	1	1306	tags=75% Kcnq4/Slc6a4/Pmch
BP	GO:00026	positive regulation of immune effector pr	230	-0.32053	-1.1695	0.0446736	0.0446736	1	2938	tags=23% Il18rap/H2-K1/Nfkbid/Cd84/F2rl1/Tnfsf13/Cxcl1/Dhx58/Tbx21/Btk/Cd40/[illegible]
BP	GO:00605	diaphragm development	8	0.822173	1.47551	0.04500324	0.04500324	1	2792	tags=50% Tcf21/Wt1/Gata4/Stra6
MF	GO:00051	insulin-like growth factor receptor bindin	14	0.749405	1.50184	0.04512715	0.04512715	1	1453	tags=21% Ins2/Ren1/Igf2
MF	GO:00081	sulfotransferase activity	46	0.580446	1.41971	0.04521277	0.04521277	1	5129	tags=33% Sult1c2/Sult1c1/Sult6b1/Chst4/Chst8/Gal3st2c/Sult6b2/Gal3st2b/Sult2b1/[illegible]
MF	GO:00302	protein tyrosine kinase activator activity	15	-0.64836	-1.45112	0.04531598	0.04531598	1	4105	tags=47% Alkal2/Dgkq/Angpt4/Nrg1/Grem1/Ghrl/Ccl5
BP	GO:00326	interleukin-21 production	3	0.921533	1.39643	0.04545455	0.04545455	1	552	tags=33% Il6
BP	GO:00140	regulation of phosphatidylinositol 3-kina	100	-0.38038	-1.23861	0.04562237	0.04562237	1	4551	tags=30% Ube3a/Sema4d/Flt1/Pdgfb/Fn1/Myoc/Cbl/Nkx3-1/Tek/F2r/Fshr/Ptpn13/[illegible]
BP	GO:00093	amine biosynthetic process	38	0.598067	1.40977	0.04583333	0.04583333	1	704	tags=11% Snca/Ldc1/Amd2/Kl
BP	GO:00424	cellular biogenic amine biosynthetic proc	38	0.598067	1.40977	0.04583333	0.04583333	1	704	tags=11% Snca/Ldc1/Amd2/Kl
MF	GO:00469	TAP binding	12	-0.69561	-1.48355	0.04635367	0.04635367	1	2932	tags=58% H2-K1/H2-D1/H2-Q4/Tap1/H2-Q7/H2-Q1/H2-Q6
BP	GO:00163	female meiosis chromosome segregation	7	0.831555	1.46512	0.04635762	0.04635762	1	2942	tags=71% Ttk/Sycp3/Plk1/Ncaph/Top2a
BP	GO:00510	chiasma assembly	7	0.830777	1.46375	0.04635762	0.04635762	1	2760	tags=43% Sycp1/Sycp3/Rnf212
BP	GO:00019	regulation of leukocyte mediated cytotoxi	86	-0.40648	-1.29244	0.04656916	0.04656916	1	2938	tags=37% Il18rap/H2-K1/Lgals9/F2rl1/Cxcl1/Tyrobp/H2-T22/H2-D1/Slamf6/Lag3/Itgam/[illegible]
BP	GO:00094	response to xenobiotic stimulus	76	0.527498	1.38969	0.04665826	0.04665826	1	1914	tags=17% Ugt1a6b/Cyp2b23/Nr1i2/Sult1c1/Cyp2j11/Fmo2/Slco1a6/Cyp2b10/Cyp2c55/[illegible]
BP	GO:00197	antifungal humoral response	9	-0.77653	-1.5478	0.04705513	0.04705513	1	892	tags=22% Camp/Ltf
BP	GO:00508	negative regulation of B cell receptor sig	6	-0.81235	-1.46761	0.04718912	0.04718912	1	1290	tags=33% Cd22/Fcgr2b
BP	GO:00706	transepithelial ammonium transport	3	0.918393	1.39168	0.04727273	0.04727273	1	1406	tags=67% Rhbg/Rhcg
BP	GO:00019	positive regulation of cytokine-mediated	48	-0.48794	-1.4148	0.04754731	0.04754731	1	4284	tags=33% Il7/Ripk1/Laptm5/Casp4/Ticam2/Edn1/Irgm1/Irf7/Parp9/Zbp1/Ikbke/Trem2/[illegible]
BP	GO:00977	sperm motility	105	0.49747	1.34646	0.04756098	0.04756098	1	4672	tags=43% Cfap157/Tekt3/Drc7/Wt1/Tmprss12/Wfdc6a/Hoatz/Nme8/Cfap206/Spag16/[illegible]
BP	GO:19046	regulation of somatic stem cell populatio	9	-0.77442	-1.54359	0.04782024	0.04782024	1	1638	tags=33% Pax2/Pax8/Hnf1b
BP	GO:00024	T cell mediated immunity	113	-0.38735	-1.27346	0.0479212	0.0479212	1	2938	tags=29% Il18rap/H2-K1/Ctsh/Was/Tbx21/Emp2/Icam1/Hmgb1/H2-T22/H2-D1/Tnfrsf1b/[illegible]
MF	GO:00308	beta-2-microglobulin binding	12	-0.69381	-1.47973	0.04796593	0.04796593	1	2932	tags=58% H2-K1/H2-D1/H2-Q4/H2-Q7/H2-T23/H2-Q1/H2-Q6
BP	GO:00354	cellular response to interferon-alpha	12	-0.69412	-1.48038	0.04796593	0.04796593	1	1184	tags=33% Ifit2/Ifi204/Ifit3/Ifit
BP	GO:20007	negative regulation of peptidyl-lysine ace	22	0.661808	1.43868	0.04804805	0.04804805	1	5449	tags=36% Snca/Mex3/Atg7/Brca1/Foxp3/Zfp451/Hint2/Pih1d1
BP	GO:00022	adaptive immune response	400	-0.30002	-1.14084	0.04811399	0.04811399	1	2954	tags=22% Ccr6/Il18rap/H2-K1/Cd79b/Il18bp/Nfkbid/Iglll1/Prdm1/Unc93b1/Cd84/C3ar1/[illegible]
MF	GO:00450	G protein-coupled purinergic nucleotide r	11	-0.72774	-1.51711	0.04812693	0.04812693	1	1621	tags=36% P2ry14/P2ry10/P2ry4/Gpr171
BP	GO:00461	negative regulation of vitamin metabolic	6	-0.80969	-1.4628	0.04865232	0.04865232	1	2272	tags=50% Gfi1/Cyp27b1/Akr1c18
BP	GO:00326	interleukin-12 production	53	-0.4509	-1.33413	0.04877201	0.04877201	1	4693	tags=36% Irf8/Mast2/Cd36/Tlr4/Il23a/Syk/Flt3/Laptm5/Rel/Cmklr1/Prkcd/Unc93b1/Cd40/[illegible]
BP	GO:00230	signal transduction involved in regulation	18	0.688885	1.45032	0.04892966	0.04892966	1	3416	tags=33% Gata4/Msx1/Cer1/Epcam/Msx2/Fgf8
BP	GO:00459	negative regulation of dopamine metaboli	4	0.887768	1.41926	0.04895105	0.04895105	1	46	tags=25% Snca
MF	GO:00038	3-beta-hydroxy-delta5-steroid dehydroge	6	0.837124	1.42939	0.04906937	0.04906937	1	1064	tags=33% Hsd3b6/Hsd3b3
BP	GO:00607	spongiotrophoblast cell proliferation	3	0.91319	1.38379	0.04909091	0.04909091	1	1453	tags=67% Tex19.1/Igf2
BP	GO:00607	cell proliferation involved in embryonic p	3	0.91319	1.38379	0.04909091	0.04909091	1	1453	tags=67% Tex19.1/Igf2
BP	GO:00902	spongiotrophoblast layer developmental g	3	0.91319	1.38379	0.04909091	0.04909091	1	1453	tags=67% Tex19.1/Igf2
BP	GO:20001	negative regulation of female gonad deve	3	0.913303	1.38396	0.04909091	0.04909091	1	1866	tags=67% Wt1/Nr5a1

r2/Ighv1-69/Nos2/Irgm2/Tlr1/Oas2/Trim75/Trim30b/Tlr8/Sh2d1b2/Nlrc5/Clec4e/Trim34a/Gbp4/Iglc3/Ccl5/Trim30d/Klrk1/Trim5/Slamf7/Ifi211/Sp110/Naip6/Ear2/Trim12a/Nlrp1a/Ltf

d22/Plg/Lhcgr/Icos/Flt3l/Azgp1/Il2rg/Klrc1/Tnf/Cd8b1/Cxcl9/Dsg1a/Klrb1c/Aqp2/Klrc1/Rtbdn/F2/Fcgr2b/Adam25/Ighv1-69/Pdcd1lg2/Msr1/Cd3g/Prn/Tlr8/Skint2/Lilrb4a/H2-Q1/Gfra3/H2-Q6/Fcgr4/Cxcl10/Iglc3/Klrk1/Slamf7/Skint3/Slc4a1/Gm5741/Cd69/Cxcr6/Prnd

gr2b/Ighv1-69/Myb/Oas2/Tlr8/Lilrb4a/H2-Q1/Sh2d1b2/Sucnr1/H2-Q6/Clec4e/Trim34a/Gbp4/Gzmb/Cxcl10/Iglc3/Ccl5/Cd300lb/Klrk1/Slfn8/Trim12

/Ccl5/Cd300lb/Gzma/Klrk1/Trav5d-4/Ifi211/Sp110/Naip6/Nlrp1a/Ltf/Epx/Pdc
Oprd1/Adgrf2/Htr2b/Hcar2/Lhcgr/Vmn1r89/Npy4r/Avpr2/Ghrhr/Pth2r/Gpr55/Olfr1/Vmn2r28/Vmn2r101/Lgr6/Vmn2r55/Olfr56/Gpr82/Adrb3/Hpgd/Gprc5d/Fpr2/Vmn2r36/Agtr1b/Oxgr1/Olfr488/Vmn2r50/Vmn1r17/Vmn2r44/Sucnr1/P2ry4/Olfr5/Gpr119/Gpr171/Vmn1r49/Vmn2r74/Vmn2r29/C5ar2/Cxc

Q6/Clec4e/Oscar/Fcgr4/Iglc3/Klrk1/C5ar2/Skint3/Ifi211/Fgl1

2-T23/Ifih1/Htr2b/Alox8/Nlrc4/Isg15/Ccl2/Ifi204/Clec9a/Ly9/Clec7a/Klrc1/Rsad2/Camp/Tnf/Tarm1/Pglyrp1/Mndal/Nepn/F2/Fcgr2b/Pdcd1lg2/Nos2/Tlr1/Psg22/Tlr8/Skint2/Lilra5/Nlrp12/Tigit/Clec4e/Gbp4/Ccl5/Klrk1/C5ar2/Skint3/Ifi211/Tnfrsf8/Nlrp1a/Ltf/Epx
66/Olfr676/Olfr165/Olfr780/Olfr566/Olfr976/Olfr1034/Olfr267/Olfr895/Olfr827/Olfr117/Azgp1/Olfr959/Olfr1/Olfr1218/Olfr1384/Olfr176/Olfr73/Olfr907/Olfr1512/Olfr307/Olfr1391/Olfr1043/Olfr293
/Olfr676/Olfr165/Olfr780/Olfr566/Olfr976/Olfr1034/Olfr267/Olfr895/Olfr827/Olfr117/Olfr959/Olfr1/Olfr1218/Olfr1384/Olfr176/Olfr73/Olfr907/Olfr1512/Olfr307/Olfr1391/Olfr1043/Olfr293

/Olfr676/Olfr165/Olfr780/Olfr566/Olfr976/Olfr1034/Olfr267/Olfr895/Olfr827/Olfr117/Olfr959/Olfr1/Olfr1218/Olfr1384/Olfr176/Olfr73/Olfr907/Olfr1512/Olfr307/Olfr1391/Olfr1043/Olfr293/Olfr1

shd1/Met/Iqcg/Saxo2/Rp1/Casc1/Ttc29/Iqub/Odf3/Pde6g/Efhc1/B9d1/Dnah5/Gapdhs/Ush2a/Akap3/Ush1g/Ttll2/Tssk1/Ccdc96/Fhdc1/Spatc1l/Dnah10/Gnat2/Cep131/Pramel1/Ace/Scnn1a/Tmem107/Spef2/Cd24a/Prcd/Cfap298/Fank1/Ano2/Lrrc56/Rsph3a/1700029J07Rik/Rilp/Stard10/Cfap58/Ezr/Pde6b/Ga

M3/Trim30a/Btnl2/Ccl3/Fgr/B2m/Tnfrsf14/Il2/H2-T23/Ifih1/Htr2b/Fabp4/Alox8/Nlrc4/Isg15/Ccl2/Ifi204/Clec9a/Avpr2/Ly9/Clec7a/Klrc1/Rsad2/Nlrp1b/Camp/Tnf/Tarm1/Pglyrp1/Mndal/Nepn/F2/Fcgr2b/Pdcd1lg2/Nos2/Tlr1/Psg22/Tlr8/Skint2/Lilrb4a/Lilra5/Nlrp12/Sucnr1/Tig

2/Fcgr2b/Irgm2/Tlr8/Lilra5/Sh2d1b2/Nlrp12/Nlrc5/Sucnr1/Cst7/Gbp4/Aoah/Ccl5/Klrk1/Ifi211/Pdc

Uqcrh/Ndufb8/Mrpl22/Ndufa11/Mrpl10/Mrps30/Uqcr10/Mrpl18/Mrpl4/Mrpl37/Tomm5/Dnajc11/Etfa/Mrps10/Sdhd/Ndufb7/Mrpl45/Ndufa5/Mrps21/Mrpl13/Uqcrb/Mrps5/Polg2/Ndufb4/Ndufa6/Mrps18c/Trmt10c/Tomm40/Mrps36/Mrpl20/Dnajc15/Tomm40l/Mrps6/Ndufs2/ND1/Ndufv2/Ndufb9/Sdhc/Immp1l/Ndufa7/Mrpl36/Ndufs3/Sdhb/Apoo/Mt

Supplement Table S3. The list of "high prediction score" mutations

ID	Location	Allele	Consequence	IMPACT	SYMBOL	Gene	Feature_ty	Feature	BIOTYPE
rs230570	1:994091	T	frameshift_vari	HIGH	Mcmdc2	ENSMUSG	Transcript	ENSMUS	protein_coding
rs262842	1:582106	T	frameshift_vari	HIGH	Aox4	ENSMUSG	Transcript	ENSMUS	protein_coding
rs251386	1:788181	-	frameshift_vari	HIGH	Kcne4	ENSMUSG	Transcript	ENSMUS	protein_coding
rs229447	1:860245	C	frameshift_vari	HIGH	Spata3	ENSMUSG	Transcript	ENSMUS	protein_coding
rs229447	1:860245	C	frameshift_vari	HIGH	Spata3	ENSMUSG	Transcript	ENSMUS	protein_coding
rs260403	1:153220	-	frameshift_vari	HIGH	Lamc1	ENSMUSG	Transcript	ENSMUS	protein_coding
rs245925	1:173270	A	frameshift_vari	HIGH	Olfr418	ENSMUSG	Transcript	ENSMUS	protein_coding
rs245925	1:173270	A	frameshift_vari	HIGH	Olfr418	ENSMUSG	Transcript	ENSMUS	protein_coding
rs241718	10:22148	-	frameshift_vari	HIGH	E030030I06Rik	ENSMUSG	Transcript	ENSMUS	protein_coding
rs241718	10:22148	-	frameshift_vari	HIGH	E030030I06Rik	ENSMUSG	Transcript	ENSMUS	protein_coding
rs262576	10:22148	-	frameshift_vari	HIGH	E030030I06Rik	ENSMUSG	Transcript	ENSMUS	protein_coding
rs262576	10:22148	-	frameshift_vari	HIGH	E030030I06Rik	ENSMUSG	Transcript	ENSMUS	protein_coding
rs232510	10:40603	-	frameshift_vari	HIGH	Slc22a16	ENSMUSG	Transcript	ENSMUS	protein_coding
rs232510	10:40603	-	frameshift_vari	HIGH	Slc22a16	ENSMUSG	Transcript	ENSMUS	protein_coding
rs260532	10:82652	TG	frameshift_vari	HIGH	Glt8d2	ENSMUSG	Transcript	ENSMUS	protein_coding
rs234015	10:88485	-	frameshift_vari	HIGH	Chpt1	ENSMUSG	Transcript	ENSMUS	protein_coding
rs247050	10:94293	G	frameshift_vari	HIGH	Gm4792	ENSMUSG	Transcript	ENSMUS	protein_coding
rs261855	11:66056	-	frameshift_vari	HIGH	Nacad	ENSMUSG	Transcript	ENSMUS	protein_coding
rs217059	11:18884	AAAAA	frameshift_vari	HIGH	Meis1	ENSMUSG	Transcript	ENSMUS	protein_coding
rs231892	11:32031	A	frameshift_vari	HIGH	Nsg2	ENSMUSG	Transcript	ENSMUS	protein_coding
rs241784	11:51651	G	frameshift_vari	HIGH	D930048N14Rik	ENSMUSG	Transcript	ENSMUS	protein_coding
rs241784	11:51651	G	frameshift_vari	HIGH	D930048N14Rik	ENSMUSG	Transcript	ENSMUS	protein_coding
rs224358	11:59161	AG	frameshift_vari	HIGH	Iba57	ENSMUSG	Transcript	ENSMUS	protein_coding
rs224358	11:59161	AG	frameshift_vari	HIGH	Iba57	ENSMUSG	Transcript	ENSMUS	protein_coding
rs251449	11:70940	T	frameshift_vari	HIGH	Rabep1	ENSMUSG	Transcript	ENSMUS	protein_coding
rs229784	11:72195	-	frameshift_vari	HIGH	4933427D14Rik	ENSMUSG	Transcript	ENSMUS	protein_coding
rs238372	11:72195	CCCCAG	frameshift_vari	HIGH	4933427D14Rik	ENSMUSG	Transcript	ENSMUS	protein_coding
rs225446	11:72309	GCTGGC	frameshift_vari	HIGH	Xaf1	ENSMUSG	Transcript	ENSMUS	protein_coding
rs224369	11:10231	C	frameshift_vari	HIGH	Ubtf	ENSMUSG	Transcript	ENSMUS	protein_coding
rs217001	11:10310	GACTG	frameshift_vari	HIGH	Acbd4	ENSMUSG	Transcript	ENSMUS	protein_coding
rs217001	11:10310	GACTG	frameshift_vari	HIGH	Acbd4	ENSMUSG	Transcript	ENSMUS	protein_coding
rs217001	11:10310	GACTG	frameshift_vari	HIGH	Acbd4	ENSMUSG	Transcript	ENSMUS	protein_coding
rs217001	11:10310	GACTG	frameshift_vari	HIGH	Acbd4	ENSMUSG	Transcript	ENSMUS	protein_coding
rs264572	11:10941	G	frameshift_vari	HIGH	9930022D16Rik	ENSMUSG	Transcript	ENSMUS	protein_coding
rs221497	11:11665	-	frameshift_vari	HIGH	Prcd	ENSMUSG	Transcript	ENSMUS	protein_coding
rs215748	12:24708	-	frameshift_vari	HIGH	Rrm2	ENSMUSG	Transcript	ENSMUS	protein_coding
rs226317	12:75722	-	frameshift_vari	HIGH	Sgpp1	ENSMUSG	Transcript	ENSMUS	protein_coding
rs250615	12:98747	G	frameshift_vari	HIGH	Zc3h14	ENSMUSG	Transcript	ENSMUS	protein_coding
rs242560	12:10426	-	frameshift_vari	HIGH	Serpina3i	ENSMUSG	Transcript	ENSMUS	protein_coding
rs249591	13:21484	A	frameshift_vari	HIGH	Zkscan4	ENSMUSG	Transcript	ENSMUS	protein_coding
rs249591	13:21484	A	frameshift_vari	HIGH	Zkscan4	ENSMUSG	Transcript	ENSMUS	protein_coding
rs264679	13:22524	-	frameshift_vari	HIGH	Vmn1r203	ENSMUSG	Transcript	ENSMUS	protein_coding
rs264679	13:22524	-	frameshift_vari	HIGH	Vmn1r203	ENSMUSG	Transcript	ENSMUS	protein_coding
rs264679	13:22524	-	frameshift_vari	HIGH	Vmn1r203	ENSMUSG	Transcript	ENSMUS	protein_coding
rs255414	13:22883	CA	frameshift_vari	HIGH	Vmn1r212	ENSMUSG	Transcript	ENSMUS	protein_coding
rs255863	13:38022	T	frameshift_vari	HIGH	Cage1	ENSMUSG	Transcript	ENSMUS	protein_coding
rs254220	13:67408	TGAG	frameshift_vari	HIGH	Zfp459	ENSMUSG	Transcript	ENSMUS	protein_coding
rs241993	13:67792	T	frameshift_vari	HIGH	Zfp493	ENSMUSG	Transcript	ENSMUS	protein_coding
rs225890	13:11945	-	frameshift_vari	HIGH	Paip1	ENSMUSG	Transcript	ENSMUS	protein_coding
rs215802	14:29021	-	frameshift_vari	HIGH	Lrtm1	ENSMUSG	Transcript	ENSMUS	protein_coding
rs262913	14:54648	-	frameshift_vari	HIGH	Gm17606	ENSMUSG	Transcript	ENSMUS	protein_coding
rs264828	14:54976	-	frameshift_vari	HIGH	Gm17428	ENSMUSG	Transcript	ENSMUS	protein_coding
rs827433	14:56019	-	frameshift_vari	HIGH	Mcpt1	ENSMUSG	Transcript	ENSMUS	protein_coding
rs249051	14:79213	C	frameshift_vari	HIGH	Zfp957	ENSMUSG	Transcript	ENSMUS	protein_coding
rs249051	14:79213	C	frameshift_vari	HIGH	Zfp957	ENSMUSG	Transcript	ENSMUS	protein_coding
rs262301	15:82390	TGGG	frameshift_vari	HIGH	Cyp2d11	ENSMUSG	Transcript	ENSMUS	protein_coding
rs246193	16:52445	-	frameshift_vari	HIGH	Alg1	ENSMUSG	Transcript	ENSMUS	protein_coding
rs246193	16:52445	-	frameshift_vari	HIGH	Alg1	ENSMUSG	Transcript	ENSMUS	protein_coding
rs231313	16:58824	A	frameshift_vari	HIGH	Olfr175	ENSMUSG	Transcript	ENSMUS	protein_coding
rs231313	16:58824	A	frameshift_vari	HIGH	Olfr175	ENSMUSG	Transcript	ENSMUS	protein_coding
rs249006	17:23945	-	frameshift_vari	HIGH	Sbp	ENSMUSG	Transcript	ENSMUS	protein_coding

rs223197	17:29379	CTGA	frameshift_vari	HIGH	Fgd2	ENSMUSG	Transcript	ENSMUS	protein_coding
rs223197	17:29379	CTGA	frameshift_vari	HIGH	Fgd2	ENSMUSG	Transcript	ENSMUS	protein_coding
rs229596	17:29615	C	frameshift_vari	HIGH	Rnf8	ENSMUSG	Transcript	ENSMUS	protein_coding
rs224819	17:35893	CCCAAC	frameshift_vari	HIGH	2310061I04Rik	ENSMUSG	Transcript	ENSMUS	protein_coding
rs219270	17:36164	TT	frameshift_vari	HIGH	Gm8909	ENSMUSG	Transcript	ENSMUS	protein_coding
rs247881	17:74635	-	frameshift_vari	HIGH	Birc6	ENSMUSG	Transcript	ENSMUS	protein_coding
rs252459	18:70240	C	frameshift_vari	HIGH	Dynap	ENSMUSG	Transcript	ENSMUS	protein_coding
rs265927	18:78350	A	frameshift_vari	HIGH	Gm6133	ENSMUSG	Transcript	ENSMUS	protein_coding
rs265927	18:78350	GA	frameshift_vari	HIGH	Gm6133	ENSMUSG	Transcript	ENSMUS	protein_coding
rs265927	18:78350	GAAA	frameshift_vari	HIGH	Gm6133	ENSMUSG	Transcript	ENSMUS	protein_coding
rs255102	19:45797	CACGCA	frameshift_vari	HIGH	Kcnip2	ENSMUSG	Transcript	ENSMUS	protein_coding
rs251085	2:313894	T	frameshift_vari	HIGH	Hmcn2	ENSMUSG	Transcript	ENSMUS	protein_coding
rs241579	2:350432	-	frameshift_vari	HIGH	Hc	ENSMUSG	Transcript	ENSMUS	protein_coding
rs228326	2:101797	-	frameshift_vari	HIGH	Prr5l	ENSMUSG	Transcript	ENSMUS	protein_coding
rs239279	2:111329	-	frameshift_vari	HIGH	Olfr1281	ENSMUSG	Transcript	ENSMUS	protein_coding
rs239279	2:111329	-	frameshift_vari	HIGH	Olfr1281	ENSMUSG	Transcript	ENSMUS	protein_coding
rs247309	2:121014	-	frameshift_vari	HIGH	Ccndbp1	ENSMUSG	Transcript	ENSMUS	protein_coding
rs247309	2:121014	-	frameshift_vari	HIGH	Ccndbp1	ENSMUSG	Transcript	ENSMUS	protein_coding
rs223637	2:125154	C	frameshift_vari	HIGH	Slc12a1	ENSMUSG	Transcript	ENSMUS	protein_coding
rs223637	2:125154	C	frameshift_vari	HIGH	Slc12a1	ENSMUSG	Transcript	ENSMUS	protein_coding
rs223637	2:125154	C	frameshift_vari	HIGH	Slc12a1	ENSMUSG	Transcript	ENSMUS	protein_coding
rs219714	2:152686	-	frameshift_vari	HIGH	H13	ENSMUSG	Transcript	ENSMUS	protein_coding
rs242513	2:164502	-	frameshift_vari	HIGH	Pigt	ENSMUSG	Transcript	ENSMUS	protein_coding
rs216130	2:164502	G	frameshift_vari	HIGH	Pigt	ENSMUSG	Transcript	ENSMUS	protein_coding
rs214958	2:164597	CC	frameshift_vari	HIGH	Wfdc8	ENSMUSG	Transcript	ENSMUS	protein_coding
rs226720	3:196104	TAAAAA	frameshift_vari	HIGH	1700064H15Rik	ENSMUSG	Transcript	ENSMUS	protein_coding
rs226994	3:364818	C	frameshift_vari	HIGH	1810062G17Rik	ENSMUSG	Transcript	ENSMUS	protein_coding
rs243754	3:936516	CT	frameshift_vari	HIGH	Tdpoz2	ENSMUSG	Transcript	ENSMUS	protein_coding
rs245839	3:945620	-	frameshift_vari	HIGH	Snx27	ENSMUSG	Transcript	ENSMUS	protein_coding
rs263506	3:122961	AG	frameshift_vari	HIGH	Usp53	ENSMUSG	Transcript	ENSMUS	protein_coding
rs246594	3:138941	-	frameshift_vari	HIGH	Rap1gds1	ENSMUSG	Transcript	ENSMUS	protein_coding
rs260910	4:340409	AA	frameshift_vari	HIGH	Spaca1	ENSMUSG	Transcript	ENSMUS	protein_coding
rs220924	4:634317	AACGT	frameshift_vari	HIGH	Whrn	ENSMUSG	Transcript	ENSMUS	protein_coding
rs247298	4:112773	-	frameshift_vari	HIGH	Skint10	ENSMUSG	Transcript	ENSMUS	protein_coding
rs239581	4:114244	T	frameshift_vari	HIGH	Skint11	ENSMUSG	Transcript	ENSMUS	protein_coding
rs239581	4:114244	T	frameshift_vari	HIGH	Skint11	ENSMUSG	Transcript	ENSMUS	protein_coding
rs261554	4:134279	TGTTTT	frameshift_vari	HIGH	Pdik1l	ENSMUSG	Transcript	ENSMUS	protein_coding
rs221612	4:141845	A	frameshift_vari	HIGH	Ctrc	ENSMUSG	Transcript	ENSMUS	protein_coding
rs221612	4:141845	A	frameshift_vari	HIGH	Ctrc	ENSMUSG	Transcript	ENSMUS	protein_coding
rs233824	4:143699	-	frameshift_vari	HIGH	Gm13089	ENSMUSG	Transcript	ENSMUS	protein_coding
rs387335	4:143851	T	frameshift_vari	HIGH	Gm13103	ENSMUSG	Transcript	ENSMUS	protein_coding
rs387335	4:143851	T	frameshift_vari	HIGH	Gm13103	ENSMUSG	Transcript	ENSMUS	protein_coding
rs387335	4:143851	T	frameshift_vari	HIGH	Gm13103	ENSMUSG	Transcript	ENSMUS	protein_coding
rs235441	5:252201	-	frameshift_vari	HIGH	Galntl5	ENSMUSG	Transcript	ENSMUS	protein_coding
rs235441	5:252201	-	frameshift_vari	HIGH	Galntl5	ENSMUSG	Transcript	ENSMUS	protein_coding
rs248422	14:48446	C	frameshift_vari	HIGH	Tmem260	ENSMUSG	Transcript	ENSMUS	protein_coding
rs247033	5:310875	GTGAT	frameshift_vari	HIGH	Slc30a3	ENSMUSG	Transcript	ENSMUS	protein_coding
rs228755	5:670976	C	frameshift_vari	HIGH	Phox2b	ENSMUSG	Transcript	ENSMUS	protein_coding
rs217195	5:145002	TCTG	frameshift_vari	HIGH	Kpna7	ENSMUSG	Transcript	ENSMUS	protein_coding
rs242224	5:145377	-	frameshift_vari	HIGH	Cyp3a57	ENSMUSG	Transcript	ENSMUS	protein_coding
rs244345	6:234100	-	frameshift_vari	HIGH	Cadps2	ENSMUSG	Transcript	ENSMUS	protein_coding
rs230117	6:898454	-	frameshift_vari	HIGH	Vmn1r42	ENSMUSG	Transcript	ENSMUS	protein_coding
rs230117	6:898454	-	frameshift_vari	HIGH	Vmn1r42	ENSMUSG	Transcript	ENSMUS	protein_coding
rs230117	6:898454	-	frameshift_vari	HIGH	Vmn1r42	ENSMUSG	Transcript	ENSMUS	protein_coding
rs231995	6:903454	-	frameshift_vari	HIGH	Uroc1	ENSMUSG	Transcript	ENSMUS	protein_coding
rs243087	6:143217	GACA	frameshift_vari	HIGH	Etnk1	ENSMUSG	Transcript	ENSMUS	protein_coding
rs265722	7:369579	-	frameshift_vari	HIGH	Tsen34	ENSMUSG	Transcript	ENSMUS	protein_coding
rs263212	7:132608	-	frameshift_vari	HIGH	Zswim9	ENSMUSG	Transcript	ENSMUS	protein_coding
rs218672	7:298356	-	frameshift_vari	HIGH	Zfp940	ENSMUSG	Transcript	ENSMUS	protein_coding
rs222341	7:302806	CTAAGA	frameshift_vari	HIGH	Thap8	ENSMUSG	Transcript	ENSMUS	protein_coding
rs222341	7:302806	CTAAGA	frameshift_vari	HIGH	Thap8	ENSMUSG	Transcript	ENSMUS	protein_coding
rs263081	7:356469	-	frameshift_vari	HIGH	Pdcd5	ENSMUSG	Transcript	ENSMUS	protein_coding
rs253193	7:856197	-	frameshift_vari	HIGH	Vmn2r71	ENSMUSG	Transcript	ENSMUS	protein_coding
rs219567	7:867952	-	frameshift_vari	HIGH	Vmn2r77	ENSMUSG	Transcript	ENSMUS	protein_coding

rs219567	7:867952	-	frameshift_varia	HIGH	Vmn2r77	ENSMUSG	Transcript	ENSMUS	protein_coding
rs255443	7:902364	G	frameshift_varia	HIGH	Ccdc83	ENSMUSG	Transcript	ENSMUS	protein_coding
rs244257	7:930809	-	frameshift_varia	HIGH	Fam181b	ENSMUSG	Transcript	ENSMUS	protein_coding
rs238147	7:100326	A	frameshift_varia	HIGH	Ppme1	ENSMUSG	Transcript	ENSMUS	protein_coding
rs217008	7:102535	CAAAC	frameshift_varia	HIGH	Olfr547	ENSMUSG	Transcript	ENSMUS	protein_coding
rs257373	7:102542	-	frameshift_varia	HIGH	Olfr548-ps1	ENSMUSG	Transcript	ENSMUS	protein_coding
rs257373	7:102542	-	frameshift_varia	HIGH	Olfr548-ps1	ENSMUSG	Transcript	ENSMUS	protein_coding
rs217263	7:102804	-	frameshift_varia	HIGH	Olfr564	ENSMUSG	Transcript	ENSMUS	protein_coding
rs249885	7:102942	-	frameshift_varia	HIGH	Olfr573-ps1	ENSMUSG	Transcript	ENSMUS	protein_coding
rs249885	7:102942	-	frameshift_varia	HIGH	Olfr573-ps1	ENSMUSG	Transcript	ENSMUS	protein_coding
rs226690	7:102942	-	frameshift_varia	HIGH	Olfr573-ps1	ENSMUSG	Transcript	ENSMUS	protein_coding
rs226690	7:102942	-	frameshift_varia	HIGH	Olfr573-ps1	ENSMUSG	Transcript	ENSMUS	protein_coding
rs246729	7:103682	-	frameshift_varia	HIGH	Olfr625-ps1	ENSMUSG	Transcript	ENSMUS	protein_coding
rs246729	7:103682	-	frameshift_varia	HIGH	Olfr625-ps1	ENSMUSG	Transcript	ENSMUS	protein_coding
rs241921	7:104357	G	frameshift_varia	HIGH	Trim30b	ENSMUSG	Transcript	ENSMUS	protein_coding
rs387093	7:106927	-	frameshift_varia	HIGH	Olfr709-ps1	ENSMUSG	Transcript	ENSMUS	protein_coding
rs387093	7:106927	-	frameshift_varia	HIGH	Olfr709-ps1	ENSMUSG	Transcript	ENSMUS	protein_coding
rs262610	7:107590	-	frameshift_varia	HIGH	Olfml1	ENSMUSG	Transcript	ENSMUS	protein_coding
rs226725	7:118442	GG	frameshift_varia	HIGH	Syt17	ENSMUSG	Transcript	ENSMUS	protein_coding
rs259251	7:118510	-	frameshift_varia	HIGH	Coq7	ENSMUSG	Transcript	ENSMUS	protein_coding
rs259892	7:139216	-	frameshift_varia	HIGH	Lrrc27	ENSMUSG	Transcript	ENSMUS	protein_coding
rs223375	8:191263	TTTTTTT	frameshift_varia	HIGH	Defb34	ENSMUSG	Transcript	ENSMUS	protein_coding
rs213590	8:260324	T	frameshift_varia	HIGH	Hook3	ENSMUSG	Transcript	ENSMUS	protein_coding
rs239380	8:408989	CT	frameshift_varia	HIGH	Slc7a2	ENSMUSG	Transcript	ENSMUS	protein_coding
rs247462	8:707301	-	frameshift_varia	HIGH	Pde4c	ENSMUSG	Transcript	ENSMUS	protein_coding
rs226815	8:104182	-	frameshift_varia	HIGH	Bean1	ENSMUSG	Transcript	ENSMUS	protein_coding
rs226815	8:104182	-	frameshift_varia	HIGH	Bean1	ENSMUSG	Transcript	ENSMUS	protein_coding
rs227791	9:382921	T	frameshift_varia	HIGH	Olfr896-ps1	ENSMUSG	Transcript	ENSMUS	protein_coding
rs227791	9:382921	T	frameshift_varia	HIGH	Olfr896-ps1	ENSMUSG	Transcript	ENSMUS	protein_coding
rs254949	9:382923	-	frameshift_varia	HIGH	Olfr896-ps1	ENSMUSG	Transcript	ENSMUS	protein_coding
rs254949	9:382923	-	frameshift_varia	HIGH	Olfr896-ps1	ENSMUSG	Transcript	ENSMUS	protein_coding
rs256178	9:411614	-	frameshift_varia	HIGH	Ubash3b	ENSMUSG	Transcript	ENSMUS	protein_coding
rs250215	9:483967	AAAA	frameshift_varia	HIGH	Nxpe4	ENSMUSG	Transcript	ENSMUS	protein_coding
rs250215	9:483967	AAAAA	frameshift_varia	HIGH	Nxpe4	ENSMUSG	Transcript	ENSMUS	protein_coding
rs260243	9:572422	-	frameshift_varia	HIGH	Trcg1	ENSMUSG	Transcript	ENSMUS	protein_coding
rs264043	9:654846	-	frameshift_varia	HIGH	Spg21	ENSMUSG	Transcript	ENSMUS	protein_coding
rs220866	9:660446	AA	frameshift_varia	HIGH	Csnk1g1	ENSMUSG	Transcript	ENSMUS	protein_coding
rs220866	9:660446	AA	frameshift_varia	HIGH	Csnk1g1	ENSMUSG	Transcript	ENSMUS	protein_coding
rs265357	9:711624	-	frameshift_varia	HIGH	Aqp9	ENSMUSG	Transcript	ENSMUS	protein_coding
rs265422	9:108797	-	frameshift_varia	HIGH	Ip6k2	ENSMUSG	Transcript	ENSMUS	protein_coding
rs257011	X:136533	A	frameshift_varia	HIGH	Kir3dl1	ENSMUSG	Transcript	ENSMUS	protein_coding
rs257011	X:136533	A	frameshift_varia	HIGH	Kir3dl1	ENSMUSG	Transcript	ENSMUS	protein_coding
rs217001	11:10310	GACTG	frameshift_varia	HIGH	Acbd4	ENSMUSG	Transcript	ENSMUS	protein_coding
rs254676	12:10370	AGTG	frameshift_varia	HIGH	Nt5c1b	ENSMUSG	Transcript	ENSMUS	protein_coding
rs254676	12:10370	AGTG	frameshift_varia	HIGH	Nt5c1b	ENSMUSG	Transcript	ENSMUS	protein_coding
rs254676	12:10370	AGTG	frameshift_varia	HIGH	Nt5c1b	ENSMUSG	Transcript	ENSMUS	protein_coding
rs254676	12:10370	AGTG	frameshift_varia	HIGH	Nt5c1b	ENSMUSG	Transcript	ENSMUS	protein_coding
rs254676	12:10370	AGTG	frameshift_varia	HIGH	Nt5c1b	ENSMUSG	Transcript	ENSMUS	protein_coding
rs254676	12:10370	AGTG	frameshift_varia	HIGH	Nt5c1b	ENSMUSG	Transcript	ENSMUS	protein_coding
rs254676	12:10370	AGTG	frameshift_varia	HIGH	Nt5c1b	ENSMUSG	Transcript	ENSMUS	protein_coding
rs219063	12:24044	-	frameshift_varia	HIGH	9030624G23Rik	ENSMUSG	Transcript	ENSMUS	protein_coding
rs387836	16:32754	A	frameshift_varia	HIGH	Muc4	ENSMUSG	Transcript	ENSMUS	protein_coding
rs219270	17:36164	TT	frameshift_varia	HIGH	Gm8909	ENSMUSG	Transcript	ENSMUS	protein_coding
rs219270	17:36164	TT	frameshift_varia	HIGH	Gm8909	ENSMUSG	Transcript	ENSMUS	protein_coding
rs231270	2:909177	A	frameshift_varia	HIGH	Ptpmt1	ENSMUSG	Transcript	ENSMUS	protein_coding
rs387791	2:121518	-	frameshift_varia	HIGH	Wdr76	ENSMUSG	Transcript	ENSMUS	protein_coding
rs247118	3:135318	GT	frameshift_varia	HIGH	Slc9b2	ENSMUSG	Transcript	ENSMUS	protein_coding
rs256092	6:137842	TGGGCA	frameshift_varia	HIGH	Dera	ENSMUSG	Transcript	ENSMUS	protein_coding
rs257960	5:923631	G	frameshift_varia	HIGH	Cxcl11	ENSMUSG	Transcript	ENSMUS	protein_coding
rs214090	11:55218	-	frameshift_varia	HIGH	Xbp1	ENSMUSG	Transcript	ENSMUS	protein_coding
rs220961	7:103682	-	frameshift_varia	HIGH	Olfr625-ps1	ENSMUSG	Transcript	ENSMUS	protein_coding
rs260631	1:150993	T	frameshift_varia	HIGH	Hmcn1	ENSMUSG	Transcript	ENSMUS	protein_coding
rs260631	1:150993	T	frameshift_varia	HIGH	Hmcn1	ENSMUSG	Transcript	ENSMUS	protein_coding
rs253978	10:12817	T	frameshift_varia	HIGH	Rbms2	ENSMUSG	Transcript	ENSMUS	protein_coding

rs239353	3:417420	-	frameshift_vari	HIGH	Sclt1	ENSMUSG	Transcript	ENSMUS	protein_coding
rs218008	6:146576	T	frameshift_vari	HIGH	Ints13	ENSMUSG	Transcript	ENSMUS	protein_coding
rs216588	7:858759	-	frameshift_vari	HIGH	Vmn2r73	ENSMUSG	Transcript	ENSMUS	protein_coding
rs216588	7:858759	-	frameshift_vari	HIGH	Vmn2r73	ENSMUSG	Transcript	ENSMUS	protein_coding
rs216588	7:858759	-	frameshift_vari	HIGH	Vmn2r73	ENSMUSG	Transcript	ENSMUS	protein_coding
rs216588	7:858759	-	frameshift_vari	HIGH	Vmn2r73	ENSMUSG	Transcript	ENSMUS	protein_coding
rs216588	7:858759	-	frameshift_vari	HIGH	Vmn2r73	ENSMUSG	Transcript	ENSMUS	protein_coding
rs216588	7:858759	-	frameshift_vari	HIGH	Vmn2r73	ENSMUSG	Transcript	ENSMUS	protein_coding
rs216588	7:858759	-	frameshift_vari	HIGH	Vmn2r73	ENSMUSG	Transcript	ENSMUS	protein_coding
rs216588	7:858759	-	frameshift_vari	HIGH	Vmn2r73	ENSMUSG	Transcript	ENSMUS	protein_coding
rs234740	1:175688	-	frameshift_vari	HIGH	Chml	ENSMUSG	Transcript	ENSMUS	protein_coding
rs212845	2:121128	-	frameshift_vari	HIGH	Lcmt2	ENSMUSG	Transcript	ENSMUS	protein_coding
rs250705	1:192343	A	frameshift_vari	HIGH	Tfap2b	ENSMUSG	Transcript	ENSMUS	protein_coding
rs250705	1:192343	AA	frameshift_vari	HIGH	Tfap2b	ENSMUSG	Transcript	ENSMUS	protein_coding
rs250705	1:192343	A	frameshift_vari	HIGH	Tfap2b	ENSMUSG	Transcript	ENSMUS	protein_coding
rs250705	1:192343	AA	frameshift_vari	HIGH	Tfap2b	ENSMUSG	Transcript	ENSMUS	protein_coding
rs254981	2:119964	TGAG	frameshift_vari	HIGH	Mga	ENSMUSG	Transcript	ENSMUS	protein_coding
rs254981	2:119964	TGAG	frameshift_vari	HIGH	Mga	ENSMUSG	Transcript	ENSMUS	protein_coding
rs254981	2:119964	TGAG	frameshift_vari	HIGH	Mga	ENSMUSG	Transcript	ENSMUS	protein_coding
rs254981	2:119964	TGAG	frameshift_vari	HIGH	Mga	ENSMUSG	Transcript	ENSMUS	protein_coding
rs236730	8:107061	TAGC	frameshift_vari	HIGH	Tmed6	ENSMUSG	Transcript	ENSMUS	protein_coding
rs243387	10:10579	T	splice_acceptor	HIGH	Mettl25	ENSMUSG	Transcript	ENSMUS	protein_coding
rs227036	12:24668	-	splice_acceptor	HIGH	Cys1	ENSMUSG	Transcript	ENSMUS	protein_coding
rs236447	14:52296	TG	splice_acceptor	HIGH	Mettl3	ENSMUSG	Transcript	ENSMUS	protein_coding
rs228816	15:96587	TT	splice_acceptor	HIGH	Slc38a1	ENSMUSG	Transcript	ENSMUS	protein_coding
rs228816	15:96587	TT	splice_acceptor	HIGH	Slc38a1	ENSMUSG	Transcript	ENSMUS	protein_coding
rs228816	15:96587	TT	splice_acceptor	HIGH	Slc38a1	ENSMUSG	Transcript	ENSMUS	protein_coding
rs251670	2:122296	TGCA	splice_acceptor	HIGH	Duox2	ENSMUSG	Transcript	ENSMUS	protein_coding
rs251670	2:122296	TGCA	splice_acceptor	HIGH	Duox2	ENSMUSG	Transcript	ENSMUS	protein_coding
rs219251	2:157101	-	splice_acceptor	HIGH	Samhd1	ENSMUSG	Transcript	ENSMUS	protein_coding
rs219251	2:157101	-	splice_acceptor	HIGH	Samhd1	ENSMUSG	Transcript	ENSMUS	protein_coding
rs219251	2:157101	-	splice_acceptor	HIGH	Samhd1	ENSMUSG	Transcript	ENSMUS	protein_coding
rs216360	4:112072	-	splice_acceptor	HIGH	Skint4	ENSMUSG	Transcript	ENSMUS	protein_coding
rs243253	11:12105	-	splice_acceptor	HIGH	Sectm1b	ENSMUSG	Transcript	ENSMUS	protein_coding
rs245068	7:193685	-	splice_acceptor	HIGH	Ppp1r13l	ENSMUSG	Transcript	ENSMUS	protein_coding
rs223913	5:383002	-	splice_acceptor	HIGH	Otop1	ENSMUSG	Transcript	ENSMUS	protein_coding
rs246861	13:22855	-	splice_acceptor	HIGH	Vmn1r211	ENSMUSG	Transcript	ENSMUS	protein_coding
rs249041	10:14760	TAAA	splice_donor_va	HIGH	Nmbr	ENSMUSG	Transcript	ENSMUS	protein_coding
rs249041	10:14760	TAAA	splice_donor_va	HIGH	Nmbr	ENSMUSG	Transcript	ENSMUS	protein_coding
rs259454	10:12700	TA	splice_donor_va	HIGH	Avil	ENSMUSG	Transcript	ENSMUS	protein_coding
rs225570	13:23807	T	splice_donor_va	HIGH	Slc17a2	ENSMUSG	Transcript	ENSMUS	protein_coding
rs227181	2:177074	-	splice_donor_va	HIGH	Gm14401	ENSMUSG	Transcript	ENSMUS	protein_coding
rs248430	6:136599	TAAA	splice_donor_va	HIGH	Atf7ip	ENSMUSG	Transcript	ENSMUS	protein_coding
rs226789	7:122983	TA	splice_donor_va	HIGH	Rbbp6	ENSMUSG	Transcript	ENSMUS	protein_coding
rs226789	7:122983	TA	splice_donor_va	HIGH	Rbbp6	ENSMUSG	Transcript	ENSMUS	protein_coding
rs226789	7:122983	TA	splice_donor_va	HIGH	Rbbp6	ENSMUSG	Transcript	ENSMUS	protein_coding
rs234841	10:77132	-	splice_donor_va	HIGH	Lats1	ENSMUSG	Transcript	ENSMUS	protein_coding
rs252335	15:79741	-	splice_donor_va	HIGH	Sun2	ENSMUSG	Transcript	ENSMUS	protein_coding
rs233926	7:195083	-	splice_donor_va	HIGH	Bloc1s3	ENSMUSG	Transcript	ENSMUS	protein_coding
rs241156	7:300720	-	splice_donor_va	HIGH	Zfp82	ENSMUSG	Transcript	ENSMUS	protein_coding
rs241156	7:300720	-	splice_donor_va	HIGH	Zfp82	ENSMUSG	Transcript	ENSMUS	protein_coding
rs241156	7:300720	-	splice_donor_va	HIGH	Zfp82	ENSMUSG	Transcript	ENSMUS	protein_coding
rs241156	7:300720	-	splice_donor_va	HIGH	Zfp82	ENSMUSG	Transcript	ENSMUS	protein_coding
rs241156	7:300720	-	splice_donor_va	HIGH	Zfp82	ENSMUSG	Transcript	ENSMUS	protein_coding
rs254207	1:580591	-	splice_donor_va	HIGH	Aox1	ENSMUSG	Transcript	ENSMUS	protein_coding
rs226993	7:198576	-	splice_donor_va	HIGH	Gm19345	ENSMUSG	Transcript	ENSMUS	protein_coding
rs218993	8:114140	-	splice_donor_va	HIGH	Col4a2	ENSMUSG	Transcript	ENSMUS	protein_coding
rs232569	5:309541	-	splice_donor_va	HIGH	Abhd1	ENSMUSG	Transcript	ENSMUS	protein_coding
rs226722	6:522606	CG	splice_donor_va	HIGH	Hoxa13	ENSMUSG	Transcript	ENSMUS	protein_coding
rs256632	11:11844	-	splice_donor_va	HIGH	C1qtnf1	ENSMUSG	Transcript	ENSMUS	protein_coding
rs260345	11:11944	-	splice_donor_va	HIGH	Rnf213	ENSMUSG	Transcript	ENSMUS	protein_coding
rs260345	11:11944	-	splice_donor_va	HIGH	Rnf213	ENSMUSG	Transcript	ENSMUS	protein_coding
rs256147	12:76031	-	splice_donor_va	HIGH	Syne2	ENSMUSG	Transcript	ENSMUS	protein_coding
rs256147	12:76031	-	splice_donor_va	HIGH	Syne2	ENSMUSG	Transcript	ENSMUS	protein_coding

rs245661	15:86299	-	splice_donor_va	HIGH	Tbc1d22a	ENSMUSG	Transcript	ENSMUS	protein_coding
rs251770	2:618063	-	splice_donor_va	HIGH	Tbr1	ENSMUSG	Transcript	ENSMUS	protein_coding
rs227938	6:135198	-	splice_donor_va	HIGH	Fam234b	ENSMUSG	Transcript	ENSMUS	protein_coding
rs249261	17:25125	AGGC	stop_gained,fra	HIGH	Ptx4	ENSMUSG	Transcript	ENSMUS	protein_coding
rs249261	17:25125	AGGC	stop_gained,fra	HIGH	Ptx4	ENSMUSG	Transcript	ENSMUS	protein_coding
rs221437	8:930681	TTAG	stop_gained,fra	HIGH	Ces1b	ENSMUSG	Transcript	ENSMUS	protein_coding
rs241001	1:132229	CTCCTA	stop_gained,infi	HIGH	Gm10188	ENSMUSG	Transcript	ENSMUS	protein_coding
rs234463	13:76062	-	stop_lost,3_prin	HIGH	Arsk	ENSMUSG	Transcript	ENSMUS	protein_coding
rs234463	13:76062	-	stop_lost,3_prin	HIGH	Arsk	ENSMUSG	Transcript	ENSMUS	protein_coding
rs220140	4:834419	-	stop_lost,3_prin	HIGH	Snapc3	ENSMUSG	Transcript	ENSMUS	protein_coding

Supplement Table S4a. The list of SZH related genes

GeneSymbol	Up/down
Abca1	up
Acox1	up
Aldh1l1	up
Anp32b	up
Aqp1	up
Atp1a2	up
Bag3	up
Bbx	up
Btg1	up
Cadm1	up
Clu	up
Crybg3	up
Ctbp2	up
Ddit4	up
Dhrs3	up
Dynlt1b	up
Echdc2	up
Edn1	up
Efemp1	up
Fam107a	up
Fermt2	up
Fyn	up
Gja1	up
Gmnn	up
Gmpr	up
Gng12	up
Gramd1c	up
Hmgb2	up
Hspb1	up
Id4	up
Ifitm1	up
Ifitm1	up
Iqgap1	up
Itgb4	up
Lgals3	up
Lpl	up
Mapre1	up
Med13l	up
Mt1	up
Nacc2	up
Necap2	up
Nipbl	up
Npas3	up

Nrxn3	up
Ntrk2	up
Oxtr	up
P4ha1	up
Palld	up
Pard3	up
Pdlim5	up
Plin2	up
Plin3	up
Plod2	up
Pon2	up
Ralgds	up
Rhobtb3	up
Ryr3	up
Scrg1	up
Selenbp1	up
Serpina3a	up
Smtn	up
Sox9	up
Tgif1	up
Tiparp	up
Tmem176a	up
Tnfrsf23	up
Tob2	up
Tra2a	up
Tulp3	up
Wfs1	up
Wnk1	up
Zhx2	up
Cd99	up
Notch2	up
Il17Rb	up
Txnip	up
Baz2B	up
Dcxr	up
Pbxip1	up
Acat3	down
Arl4c	down
Atp1b3	down
Atp6v1a	down
Atp6v1d	down
Atp8a2	down
Atrnl1	down
Bbs7	down
Bcl6	down
Blvra	down

Cacnb1	down
Clcn4	down
Crhbp	down
Cyp26b1	down
Diras2	down
Dlgap1	down
Dync1li1	down
Edn3	down
Egr1	down
Ensa	down
Ets2	down
Etv5	down
Fabp3	down
Fbxo9	down
Fbxw7	down
Fhl2	down
Glce	down
Hbq1b	down
Hint1	down
Idh3a	down
Itm2a	down
Kcnk1	down
Lbh	down
Lgals8	down
Lrrc17	down
Map2k4	down
Mpped2	down
Ndufb6	down
Nell1	down
Nmu	down
Nptx2	down
Npy	down
Nr4a2	down
Olfm1	down
Penk	down
Phtf1	down
Pias2	down
Pnoc	down
Prmt8	down
Prps1	down
Prss16	down
Slc25a12	down
Slc29a1	down
Smyd2	down
Snn	down
Sulf1	down

Syngr3	down
Tac1	down
Tbc1d9	down
Timm17a	down
Tnfsf10	down
Try10	down
Ube2d2a	down
Vsnl1	down
Tpbg	down
Snx10	down
Arpp19	down
Opn3	down
Cdc42	down
Prkacb	down
Reep1	down
Mcts1	down
Pvalb	down
Lrmp	down
Sts	down
Uchl5	down
Moap1	down

Supplement Table S4b. The list of ASD related genes

GeneSymbol	Up/down
Hspb1	up
Adam12	up
Slc2a5	up
Csf3r	up
Mapkapk2	up
Palld	up
Siglece	up
Col1a1	up
Sell	up
Foxj1	up
Apoe	up
Gypc	up
Ribc1	up
C3	up
Pgghg	up
Slc9a9	up
Pik3ap1	up
Bace2	up
Glis2	up
Agtrap	up
Arid5a	up
Hck	up
Ifi205	up
Phf21b	up
S100a3	up
Cebpd	up
Zfp36	up
Ngfr	up
Sla	up
Bves	up
Sgk1	up
Irf7	up
Plin3	up
Sectm1a	up
Tmem176a	up
Trim56	up
Il21r	up
Tlr5	up
Elf1	up
Cd93	up
Itgb2	up
Bcl6	up
Alpk1	up

Tnfaip3	up
Clic1	up
Pdk4	up
Lilra5	up
Abi3	up
Prrx2	up
Ptger4	up
Wee1	up
Rgs1	up
Gadd45b	up
Tead4	up
Ucp2	up
Ptprc	up
Hmox1	up
Dtx2	up
Batf2	up
Ifit1bl2	up
Spi1	up
Zfp36l1	up
Stab1	up
Cfap53	up
Gbp1	up
Oas2	up
Trpv4	up
Thbs1	up
Srgap1	up
Cdk2	up
Gpr84	up
Gipc3	up
Il4ra	up
Tgif1	up
Tm4sf1	up
Vasp	up
Cdkn1a	up
Lcp1	up
Rhpn2	up
Nuak2	up
Fosb	up
Alas2	up
Hamp	up
Tlr2	up
Parp9	up
Hap1	up
Havcr2	up
Gm49342	up
Eya2	up

Bcl2l12	up
Nfe2	up
C1ra	up
Sox4	up
Msn	up
Dnajb1	up
Plek	up
Fgf11	up
Tgm2	up
Epha2	up
Abhd4	up
Nfatc1	up
Tagln2	up
Ezh2	up
Osmr	up
Abca1	up
Tiparp	up
Dtx3l	up
Dbx2	up
Muc1	up
Gng5	up
C3ar1	up
Efhd1	up
Adamts9	up
Cfh	up
C4b	up
Kifc1	up
Nupr1	up
Dsc2	up
Cxcl11	up
Il1rl1	up
Fcgr1	up
Cyp4f18	up
Parp14	up
Ftl1-ps1	up
Eps8l1	up
Gja4	up
Mthfd2	up
Myof	up
Samd9l	up
Helz2	up
Sphk1	up
Prkx	up
Gbp2b	up
Was	up
Lat2	up

Sh3tc1	up
S100a8	up
Lix1	up
Mcl1	up
Il6	up
Batf	up
Mvp	up
Fgfrl1	up
Vdr	up
Cd37	up
Oscar	up
Lats2	up
Gadd45g	up
Bst2	up
Sp100	up
Pygl	up
Cfi	up
Cd5l	up
Ube2c	up
Gpr4	up
Ntn1	up
Tcirg1	up
Kif1c	up
Lyl1	up
Rhbdf2	up
Rab13	up
Ifi35	up
Fam124b	up
Msln	up
Wwtr1	up
Plekha4	up
Kif20a	up
Yap1	up
Spata13	up
Rfx4	up
Pi16	up
Plac8	up
Mfng	up
Gem	up
Pard3	up
Cxcl16	up
Npl	up
Anp32b	up
Itprip	up
Ifi44	up
Hlx	up

Spocd1	up
Gm5849	up
Mmp9	up
Ctsc	up
Rit2	down
Nap1l2	down
Kcnc1	down
Slc6a15	down
Slc6a7	down
Nomo1	down
Slc10a4-ps	down
Epha4	down
Apba2	down
Frmpd4	down
Kit	down
Slc17a6	down
Gabrd	down
Ckmt1	down
Sncg	down
Cplx2	down
Kcnv1	down
Rimkla	down
Atp2b3	down
Mllt11	down
Olfm3	down
Rtn4r	down
Fkbpl	down
Sez6l2	down
Cidea	down
Sult4a1	down
Smyd2	down
Syt1	down
Cdh22	down
Glrb	down
Gpat3	down
